# Identification of the Glyceraldehyde-3-Phosphate Dehydrogenase (*GeGAPDH*) Gene Family in *Gastrodia elata* Revealing Its Response Characteristics to Low-Temperature and Pathogen Stress

**DOI:** 10.3390/plants14121866

**Published:** 2025-06-18

**Authors:** Yaxing Yan, Mei Jiang, Pengjie Han, Xiaohu Lin, Xiao Wang

**Affiliations:** 1 Hebei Key Laboratory of Crop Stress Biology, College of Agronomy and Biotechnology, Hebei Normal University of Science and Technology, Qinhuangdao 066000, China; 18149114906@163.com; 2 Analysis and Testing Center, Hebei Normal University of Science and Technology, Qinhuangdao 066000, China; 3 Shandong Engineering Research Center for Innovation and Application of General Technology for Separation of Natural Products, Shandong Analysis and Test Center, Qilu University of Technology, Jinan 250014, China; mjiang0502@163.com (M.J.); hanpengjie2023@126.com (P.H.); 4 Key Laboratory for Natural Active Pharmaceutical Constituents Research in Universities of Shandong Province, School of Pharmaceutical Sciences, Qilu University of Technology, Jinan 250014, China

**Keywords:** *Gastrodia elata*, glyceraldehyde-3-phosphate dehydrogenase, enzymatic activity, subcellular localization, stress response

## Abstract

The glyceraldehyde-3-phosphate dehydrogenase (*GAPDH*) gene plays a pivotal role in the glycolysis/gluconeogenesis process, contributing significantly to glycosyl donor synthesis, plant growth and development, and stress responses. *Gastrodia elata* Bl., a heterotrophic plant in the Orchidaceae family, has its dried tubers used as the traditional Chinese medicine. This study identified three *GeGAPDH* genes in *G. elata*, all encoding basic, stable, hydrophilic proteins. Phylogenetic analysis and subcellular localization predictions categorized *GeGAPDH1* as a plastid subtype, while *GeGAPDH2* and *GeGAPDH3* were classified as cytoplasmic subtypes. Prokaryotic expression experiments demonstrated successful expression of the GeGAPDH1 protein in *Escherichia coli*, which exhibited significant GAPDH enzymatic activity. Subcellular localization experiments showed that GeGAPDH1 was localized in the plastid. Expression analysis indicated that the three *GeGAPDH* genes were predominantly expressed in tubers. Under low-temperature stress, although the total GAPDH enzyme activity in tubers did not change significantly, the expression of *GeGAPDH1* was significantly up-regulated, while *GeGAPDH2* and *GeGAPDH3* were significantly down-regulated. This suggests that different subtypes of *GeGAPDH* may regulate cold resistance through different pathways. Upon pathogen infection, the *GeGAPDH* gene family exhibited pathogen-specific regulatory patterns. During infection by *Fusarium oxysporum*, both the expression levels of all three *GeGAPDH* genes and the total GAPDH enzyme activity in tubers increased significantly; however, *F. solani* infection induced a significant increase in total GAPDH enzyme activity without significant changes in gene expression. These results suggest that the *GeGAPDH* gene family may respond to different pathogen infections through transcriptional or translational regulation mechanisms. This study systematically identified and characterized the *GeGAPDH* gene family in *G. elata*, providing a theoretical foundation for understanding the functional differentiation of *GAPDH* in heterotrophic plants.

## 1. Introduction

*Gastrodia elata* Bl. is a heterotrophic perennial herb that forms a symbiotic relationship with fungi belonging to the genus *Gastrodia* in the Orchidaceae family. Its dried tubers are a traditional Chinese medicinal resource with significant pharmacological and economic importance. Modern pharmacological studies have demonstrated that *G. elata* exhibits various therapeutic effects, including anticonvulsant, antiseizure, sleep-improving, memory-enhancing, antidepressant, and anti-aging properties [1,2,3,4]. These findings indicate its broad application potential in pharmaceuticals, health supplements, and cosmetics. The primary active components of *G. elata* include gastrodin, parishins, and polysaccharides, which play crucial roles in antioxidation, anti-aging, neuroprotection, and immune regulation [5,6,7,8]. However, during cultivation, *G. elata* is often subjected to various biotic and abiotic stresses, which adversely affect both its yield and quality. For instance, prolonged low-temperature stress can lead to the development of black spots and, in severe cases, lead to rot and necrosis [9]. Additionally, soil-pose fungal diseases are significant threats to *G. elata* cultivation, including brown rot, black rot, rust rot, and white silk disease, all caused by pathogenic fungi [10,11,12]. Nevertheless, the molecular mechanisms underlying *G. elata*’s responses to various biotic and abiotic stresses remain poorly understood.

Glyceraldehyde-3-phosphate dehydrogenase (GAPDH) [EC: 1.2.1.12] is one of the key enzymes involved in glycolysis/gluconeogenesis and carbon fixation in the Calvin-Benson cycle. In animals, *GAPDH* is highly conserved, with only one subtype identified [13]. In contrast, plants exhibit multiple GAPDH subtypes with distinct cellular compartmentalization. For example, *Arabidopsis thaliana* contains two cytosolic subtypes, *GAPC1/GAPC2* [14], three chloroplast subtypes, *GAPA1/GAPA2/GAPB* [15], two plastid subtypes, *GAPCp1/GAPCp2* [16], and the non-phosphorylating, NADP-dependent *GAPN* [17]. *GAPC* and *GAPN* are involved in glycolysis [18], *GAPA/B* is involved in carbon fixation by the Calvin-Benson cycle [19], and *GAPCp* operates in the plastid glycolytic pathway [16]. GAPDH typically exists as a tetramer, with its subunits being highly conserved, containing two critical domains, Gp_dh_N and Gp_dh_C [20]. Moreover, many conserved amino acids may undergo post-translational modifications such as acetylation, phosphorylation, succinylation, or ubiquitination [21]. Although the *GAPDH* gene family has been extensively studied in green plants, its subcellular classification and functional differentiation may vary across species. In heterotrophic plants lacking chloroplasts, the loss of *GAPA/B* subtypes involved in the gluconeogenesis pathway may be compensated by other *GAPDH* subtypes. *G. elata* depends on *Armillaria mellea* for carbon supply, suggesting that its gluconeogenesis pathway may be different from that of photosynthetic plants.

To date, several plant *GAPDH* genes have been successfully cloned and characterized, including those from *A*. *thaliana* [22], pepper [23], tomato [24], maize [25], *Codonopsis pilosula* [26], and *Panax notoginseng* [27]. *GAPDH* is regarded not only as a model gene for studying enzyme structure and activity but also as an important reference gene for analyzing relative expression levels [28]. Additionally, research has shown that *GAPDH* genes play crucial roles in plant growth and development, as well as in responses to abiotic stresses. For example, overexpression of the watermelon gene *ClGAPC2* and the soybean gene *GmGAPDH*14 significantly enhances salt tolerance [29,30]. The wheat gene *TaGAPCp1* is involved in drought stress regulation [31], and the plastid *GAPDH* in *A. thaliana* participates in abscisic acid signaling, thereby influencing seed germination and plant growth [32]. Furthermore, *GAPDH* genes also play important roles in plant responses to biotic stresses. When exposed to the pathogen *Pseudomonas syringae*, *A. thaliana AtGAPA*, *AtGAPC*, and *AtGAPCp* regulate the accumulation of reactive oxygen species and cell death, negatively modulating disease resistance [13]; Cassava *MeGAPC* is considered a negative regulator of plant disease resistance, inhibiting autophagic activity through interactions with autophagy-related proteins [33]. Additionally, the *Brassica napus* genes *BnaGAPDH17*, *BnaGAPDH20*, *BnaGAPDH21*, and *BnaGAPDH22* exhibit sensitivity to infections by *Sclerotinia sclerotiorum* [34]. However, the expression patterns of the *GAPDH* genes in *G. elata* under various growth processes and stress conditions remain unclear.

As a heterotrophic plant, *G. elata* lacks photosynthetic carbon fixation capacity. Instead, it fully compensates for this deficiency by acquiring carbon from symbiotic fungi to support germination and growth [35]. This difference in carbon acquisition likely leads to evolutionary adaptations in glucose metabolism, especially gluconeogenesis, which may be different from that of photosynthetic plants. Although the role of *GAPDH* in plant glucose metabolism and stress response has been extensively studied, the functional differentiation of *GAPDH* in heterotrophic plants remains unclear. In this study, we systematically identified the *GeGAPDH* gene family in *G. elata* and comprehensively analyzed the structural characteristics, classification, subcellular localization and enzyme activity of *GeGAPDH* genes. In addition, we investigated the response patterns of *GeGAPDH* genes to both biotic and abiotic stresses through analyses of cis-acting elements, gene expression patterns, and enzyme activity assessments. This study reveals the functional differentiation and stress response patterns of *GAPDH* subtypes in the heterotrophic plant *G. elata*, providing new insights for understanding the function and stress adaptation of *GAPDH* genes in symbiotic plants.

## 2. Results

### 2.1. Identification and Physicochemical Characterization of the GeGAPDH Family

Through comparative analysis of homologous sequences of the *GAPDH* gene in *A. thaliana* and examination of conserved domains (Gp_dh_N and Gp_dh_C), we identified three *GAPDH* genes in the *G. elata* genome (Table S1). Based on their chromosomal distribution, these genes were designated as *GeGAPDH1*, *GeGAPDH2*, and *GeGAPDH3*, with *GeGAPDH2* and *GeGAPDH3* sharing identical amino acid sequences. Prediction of the physicochemical properties of the amino acid sequences encoded by these three genes revealed that their lengths range from 344 to 428 amino acids, molecular mass varies between 37,088.64 and 45,867.0 Da, and isoelectric points are between 7.68 and 8.51, indicating that they are basic proteins. The instability index ranged from 26.75 to 37.51, suggesting relatively high stability of these proteins within the cellular environment. The average hydrophilicity coefficient ranged from −0.01 to −0.106, with all values being negative, indicating that the GAPDH proteins from *G. elata* exhibit hydrophilic properties (Table 1). Subcellular localization predictions indicated that *GeGAPDH1* is localized in plastids, while *GeGAPDH2* and *GeGAPDH3* are localized in the cytoplasm.

### 2.2. Gene Structure and Conserved Domain Analysis of the GeGAPDH Family

**Figure 1:**
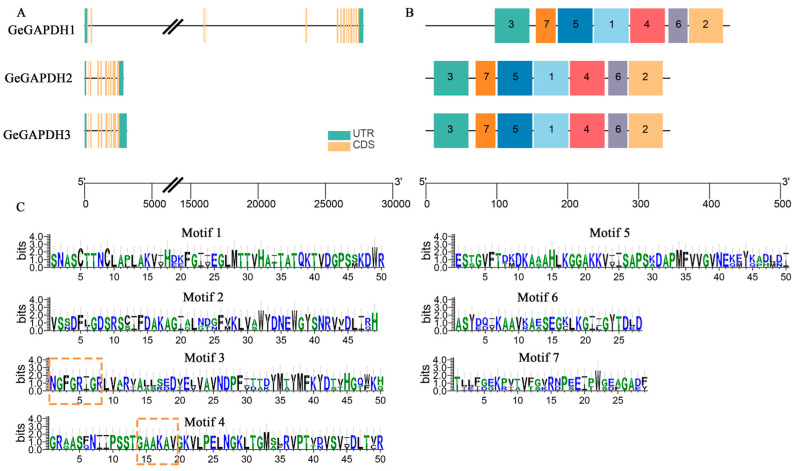
Gene structure and conserved structural domains of the *GeGAPDH* family in *G. elata*. (**A**) Gene structure of the *GeGAPDH* family of *G. elata*. (**B**) Distribution of conserved structural domains of the GeGAPDH family. (**C**) Seven conserved structural domains in the GeGAPDH family. Conserved domains are shown in yellow boxes.

Gene structures analysis revealed that *GeGAPDH1* consists of 13 introns and 14 exons, while *GeGAPDH2* and *GeGAPDH3* each contain 9 introns and 10 exons (Figure 1A). The analysis of conserved motifs in the amino acid sequences showed that all three *GeGAPDH* genes share seven conserved motifs (Figure 1B). Specifically, motifs 1, 2, 3, 4, and 5 consist of 50 amino acids each, while motifs 6 and 7 contain 28 and 29 amino acids, respectively. Motif 3 encompasses the Gp_dh_N domain (INGFGRIGR), and motif 4 contains the Gp_dh_C domain (GAAKAV) (Figure 1C). The high degree of similarity in gene structure and conserved domains between *GeGAPDH2* and *GeGAPDH3* suggests that the *GAPDH* genes in *G. elata* may have undergone gene duplication events.

### 2.3. Phylogenetic Analysis of the GAPDH Family

**Figure 2:**
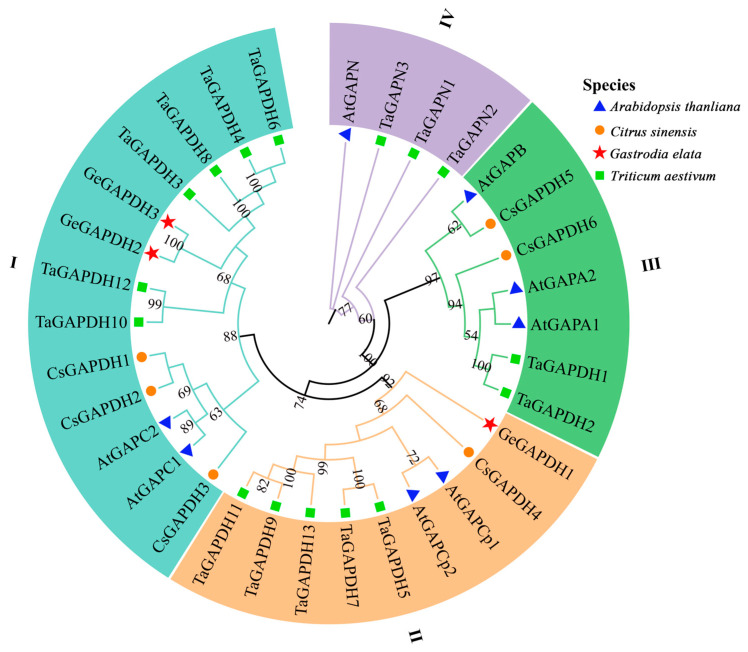
Phylogenetic tree of GAPDH family. Phylogenetic trees were constructed using 33 GAPDH protein sequences from different species. Phylogenetic analyses were performed using maximum likelihood and 1000 bootstrap replicates. The GAPDH family is divided into four subgroups (Group I–Group IV) and is distinguished by different colors. Different species are represented by different symbols.

To explore the phylogenetic relationships among GAPDH family proteins, a phylogenetic tree was constructed using three GeGAPDH proteins, six *Citrus sinensis*, and eight *A. thaliana*, and 16 *Triticum aestivum* GAPDH protein sequences [20,37]. The 33 GAPDH proteins were classified into four distinct subgroups, numbered in a counterclockwise manner. Notably, the clustering results are consistent with the previously reported phylogenetic analysis [20]. Furthermore, GAPDH proteins are typically divided into four functional subfamilies based on the established nomenclature and subcellular localization data of *A. thaliana* GAPDH. The classification of the four subgroups was determined (Figure 2): Subfamily I (GAPC), Subfamily II (GAPCp), Subfamily III (GAPA/B), and Subfamily IV (GAPN) [13,20]. Proteins in the same subgroup may perform similar functions. In *G. elata*, GeGAPDH1 was grouped into subfamily II, while GeGAPDH2 and GeGAPDH3 were clustered in subfamily I (Supplementary Materials Data S1, Figure 2). This classification is further supported by the conserved functional roles observed in homologous proteins across species. Based on phylogenetic relationships, it can be inferred that GeGAPDH1 is a member of the plastid subtype, whereas GeGAPDH2 and GeGAPDH3 belong to the cytosolic subtype.

### 2.4. Chromosomal Localization and Collinearity Analysis of the GeGAPDH Family

**Figure 3:**
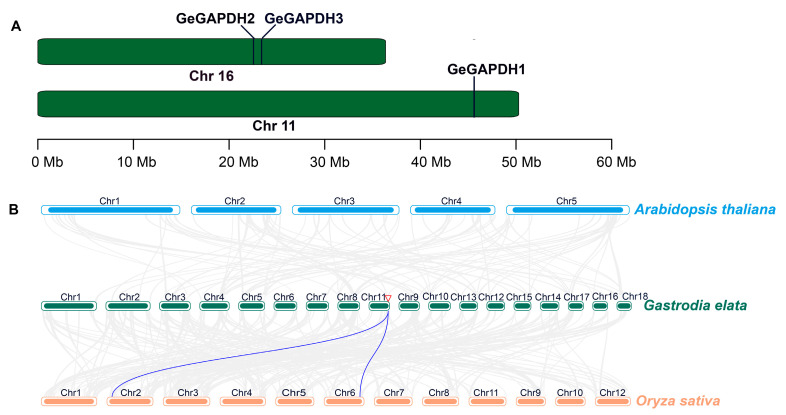
Chromosomal localization and collinearity analysis of the *GeGAPDH* family in *G. elata*. (**A**) Chromosomal localization of the *GeGAPDH* family. (**B**) Collinearity analysis of the *GAPDH* families among *A. thaliana*, *G. elata* and *O. sativa*. The red triangle is the location of the *GeGAPDH1* gene.

Genomic annotation analysis enabled a chromosomal localization map for the *GeGAPDH* family genes (Figure 3A). The results indicated that the *GeGAPDH* genes are distributed across two chromosomes, with *GeGAPDH1* located on chromosome 11 and *GeGAPDH2* and *GeGAPDH3* on chromosome 16. Additionally, collinearity analysis among the genomes of *G. elata*, *A. thalian*, and *Oriza sativa* revealed a high degree of collinearity between the *GeGAPDH* genes and the monocotyledonous plant *O. sativa*, while the collinearity with the dicotyledonous plant *A. thalian* was lower (Figure 3B). Notably, *GeGAPDH1* exhibited duplication events with two *O. sativa* genes, suggesting a shared ancestral origin and potential functional similarities.

### 2.5. Cis-Acting Element Analysis of the GeGAPDH Family

**Figure 4:**
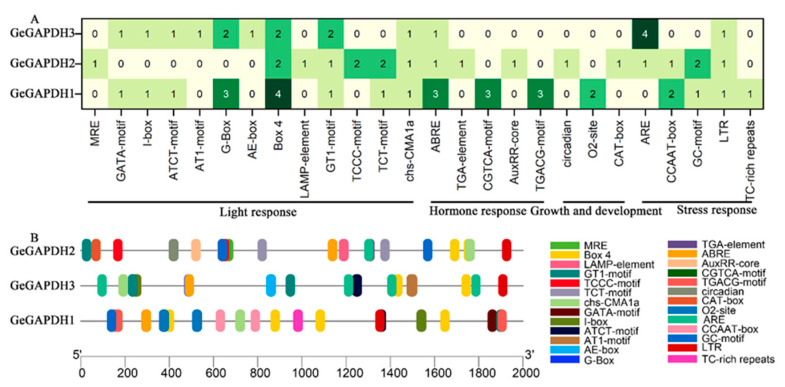
Cis-acting elements of the *GeGAPDH* family in *G. elata*. (**A**) Types of cis-acting elements of the *GeGAPDH* family. The intensity of the color represents the number of cis-acting elements. (**B**) Distribution of cis-acting elements of the *GeGAPDH* family.

Using PlantCARE (https://bioinformatics.psb.ugent.be/webtools/plantcare/html/, accessed on 23 November 2024), cis-acting elements in the upstream 2000 bp regions of all *GeGAPDH* genes were analyzed. The results revealed that the promoter regions of the three genes collectively contain 26 types of cis-acting elements. These elements were categorized based on their functions into four classes: light response, hormone response, plant growth and development, and stress response (Figure 4A). The promoter of *GeGAPDH1* contains the highest number of cis-acting elements, totaling 29, with Box4 being the most abundant at four occurrences. The promoter of *GeGAPDH2* and *GeGAPDH3* contains 20 and 18 cis-acting elements, respectively, with ARE being the most prevalent at four occurrences. Despite *GeGAPDH2* and *GeGAPDH3* sharing identical amino acid sequences, differences in the types and distributions of cis-acting elements in their promoter regions suggest that their transcriptional regulation may be governed by distinct mechanisms (Figure 4B). Furthermore, six cis-acting elements were common to all three *GeGAPDH* genes, including the light-responsive element Box4, GT1-motif, and chs-CMA1a, as well as the phytohormone response element ABRE and the stress response element LTR. These results suggest that the *GeGAPDH* gene family may play regulatory roles in the growth and development of *G. elata*, as well as in responses to light, phytohormones, and stress.

### 2.6. Interaction Analysis of GeGAPDH Proteins

**Figure 5:**
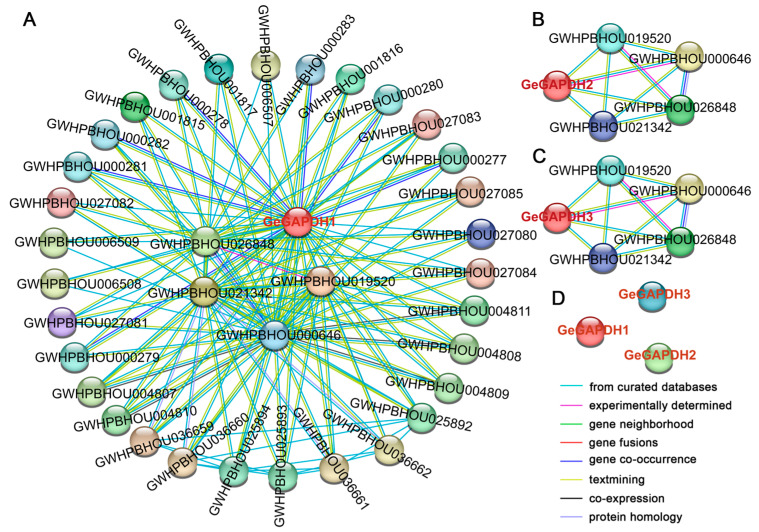
The protein–protein interactions (PPI) of GAPDH proteins in *G. elata*. (**A**–**C**) Predicted protein interaction network of three GeGAPDH proteins. (**D**) No interactions were predicted among the three GeGAPDH proteins using identical parameters. Edges represent functional associations between proteins, indicating their potential involvement in shared biological processes. Edge colors indicate different prediction types.

Figure 5 presents the predicted protein-protein interaction (PPI) networks of three GeGAPDH proteins under the highest confidence parameters (interaction score ≥ 0.900). Among them, GeGAPDH1 exhibits an extensive interaction network with 35 target proteins, while GeGAPDH2 and GeGAPDH3 display a significantly narrower interaction network, each forming interactions with only 4 proteins. All four interacting proteins of GeGAPDH2 and GeGAPDH3 are encompassed within the broader interaction network of GeGAPDH1. Under these stringent confidence thresholds, no direct interactions were detected among the three GeGAPDH proteins, suggesting that they may function independently within distinct interaction networks or require specific conditions to form complexes.

### 2.7. Enzyme Activity and Kinetics Detection of the GeGAPDH1

**Figure 6:**
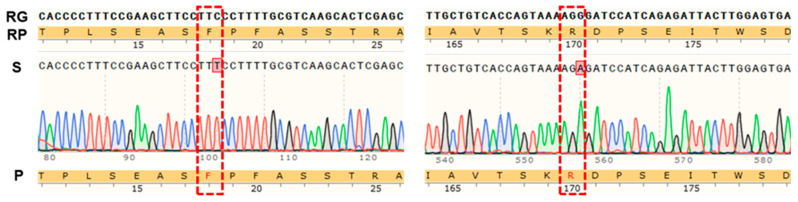
The Gene cloning results of *GeGAPDH1*. RG is the reference gene sequence, and RP is the corresponding protein sequence of the reference gene. S is the result of sequencing, and the red dashed line box is the location of the base mutation. P is the protein sequence corresponding to the sequencing result.

As a non-photosynthetic autotrophic plant, the gluconeogenesis process of *G. elata* may be different from that of photosynthetic plants. Thus, we selected the genes of plastid subtypes *GeGAPDH1* for gene cloning and enzyme activity assay to analyze the potential function of *GeGAPDH1* in gluconeogenesis. The *GeGAPDH1* gene was successfully cloned. A comparison of the sequencing results with the reference genome showed that the *GeGAPDH1* gene sequence contained synonymous mutations at the 101st (C-T) and 1535th (G-A) nucleotide positions, which did not alter the amino acid sequence (Figure 6 and Figure S1). Differences in sequences may be due to individual differences in species.

**Figure 7:**
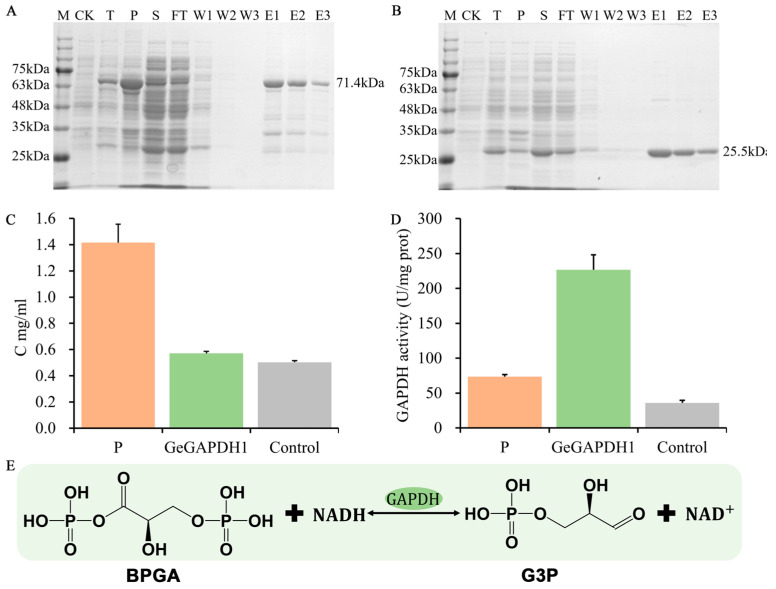
SDS-PAGE, concentration and GAPDH activity detection. (**A**) The protein expression of GeGAPDH1. (**B**) The protein expression of empty vector. M: protein marker (11~180 kDa); CK: non-induced bacterial solution; T: total protein; S: supernatant after crushing; P: precipitation after crushing; FT: flow-through after incubation binding; W1–W3, washing solution 1–3; E1–E3, protein elution solution 1–3 The predicted molecular mass of the target protein is labeled on the right side of the gel graph. (**C**) The concentration of protein P was the total protein, GeGAPDH1 was the purified protein of GeGAPDH1, and the control was the purified protein of the empty vector. *n* = 3 technical replicates. (**D**) The results of GAPDH1 activity assay. *n* = 3 technical replicates. (**E**) Reactions occurring in enzyme activity assays. In this reaction, GAPDH catalyzes BPGA and NADH to G3P and NAD^+^. The decrease of NADH measured at 340 nm can reflect the level of GAPDH activity. Consumption of 1 nmol of NADH per minute per mg of protein was defined as one unit of enzyme activity.

Subsequently, we constructed a recombinant plasmid by ligating the obtained *GeGAPDH1* gene with the expression vector pGEX-4T-1 for protein expression in *Escherichia coli*. The predicted molecular mass of the recombinant GeGAPDH1 protein was 71.4 kDa, which corresponds to the sum of the predicted protein molecular mass (45.9 kDa) and the GST tag (25.5 kDa) (Figure 7A,B). SDS-PAGE analysis confirmed the successful expression and purification of the GeGAPDH1 protein, as indicated by the band corresponding to the predicted molecular mass of the protein.

In green plants, both *GAPC* and *GAPCp* subtypes involved in glycolysis are defined as phosphorylating because they all catalyze in vitro the phosphorylation of the substrate glyceraldehyde-3-phosphate (G3P) to 1,3-bisphosphoglyceric acid (BPGA). However, the physiological role of *GAPA/B* in the Calvin-Benson cycle is in the opposite direction, catalyzing the dephosphorylation of the substrate BPGA [38]. We measured the concentration of the total protein and purified proteins using the Bradford method (Figure 7C) and assessed GAPDH enzyme activity. The results indicated that the purified GeGAPDH1 protein exhibited significantly higher GAPDH enzyme activity compared to both the total protein and the control group (empty vector-expressed protein) (Figure 7D,E).

**Figure 8:**
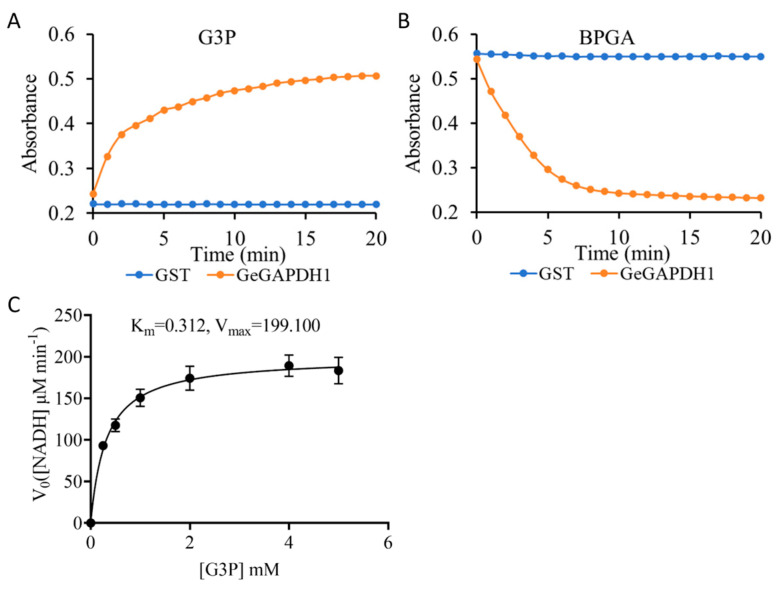
Bidirectional enzyme activity and kinetic characterization of GeGAPDH1. (**A**) The phosphorylation reaction uses G3P as a substrate to generate NADH. (**B**) The dephosphorylation reaction using BPGA as a substrate consumes NADH. (**C**) Michaelis-Menten kinetic analysis of GeGAPDH1 with varying G3P concentrations.

The bidirectional enzymatic assay showed that GeGAPDH1 possesses both NADH-producing and NADH-consuming capabilities, suggesting its bifunctional glyceraldehyde-3-phosphate dehydrogenase activity (Figure 8A,B). Kinetic analysis revealed that GeGAPDH1 exhibited a Km of 0.312 mM and Vmax of 199.100 μM NADH min^−1^ mg^−1^ (enzyme activity was defined as the production of 1 μM NADH per mg protein per minute) for its substrate G3P (Figure 8C). These kinetic parameters fall within the broad spectrum reported for GAPDH enzymes across different species [39].

### 2.8. Subcellular Localization Analysis of GeGAPDH1

**Figure 9:**
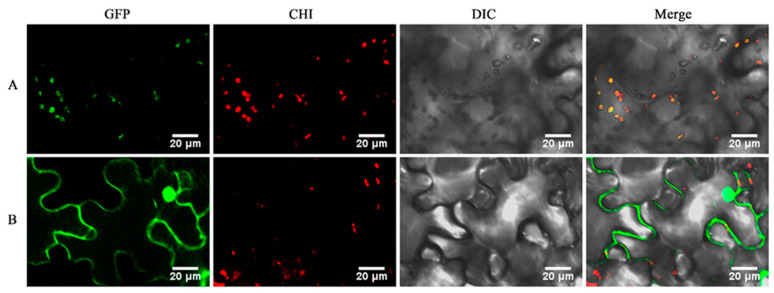
Subcellular localization of GeGAPDH1. (**A**) The fusion vector pCAMBIA1300-35S-GeGAPDH1-EGFP is transiently expressed in *N. benthamiana*. (**B**) The empty vector pCAMBIA1300-35S-EGFP is transiently expressed in *N. benthamiana*. GFP represents the green fluorescence field, CHI represents the chloroplast spontaneous fluorescence field, DIC represents the bright field, and Merge represents the superposition field. GFP field: excitation 488 nm, emission 500–550 nm; CHI field: excitation 488 nm, emission 650–750 nm. Scale bar = 20 µm.

Subcellular localization analysis revealed that the fluorescence signal from the fusion vector pCAMBIA1300-35S-GeGAPDH1-EGFP was primarily localized in plastids, exhibiting strong colocalization with the chloroplast fluorescence signal in *Nicotiana benthamiana* leaves (Figure 9A). This observation confirms that the GeGAPDH1 protein is plastid-localized, which corroborates the predicted and phylogenetic results. In contrast, the signal from the empty vector pCAMBIA1300-35S-EGFP was observable throughout the cell, with green fluorescence predominantly in the nucleus and plasma membrane, along with weaker expression in the cytoplasm (Figure 9B).

### 2.9. Expression Characteristics of the GeGAPDH Family Among Tissues

**Figure 10:**
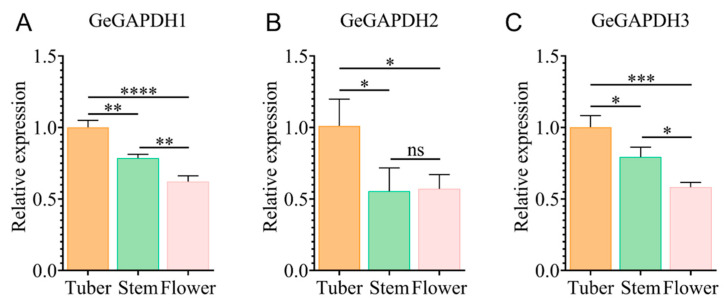
Relative expression of *GeGAPDH* genes in different tissues. The X-axis represents different tissues, and the Y-axis represents the relative expression levels normalized to the expression values of the tuber. Error bars represent the standard error. Significant differences are indicated as follows: ns, non-significant; * *p* < 0.05; ** *p* < 0.01; *** *p* < 0.001; **** *p* < 0.0001. Values represent means ± SD (*n* = 3).

Using the *GeActin* gene as an internal reference, we employed qRT-PCR to analyze the expression of *GeGAPDH* genes in various tissues. Using the tuber expression level of *GeGAPDH* genes as the reference (set to 1), we compared the gene expression levels in tubers, stems, and flowers. The analysis revealed significant differences in the expression levels of *GeGAPDH* genes among the three tissues, with all three genes showing predominant expression in tubers. Specifically, the expression levels of *GeGAPDH1*, *GeGAPDH2* and *GeGAPDH3* in stems were 0.79-, 0.55-, and 0.79-fold, respectively, compared to tubers. In flowers, the expression levels were 0.62-, 0.57-, and 0.58-fold, respectively, compared to tubers (Figure 10).

### 2.10. Expression Characteristics of the GeGAPDH Family Under Low-Temperature Stress

**Figure 11:**
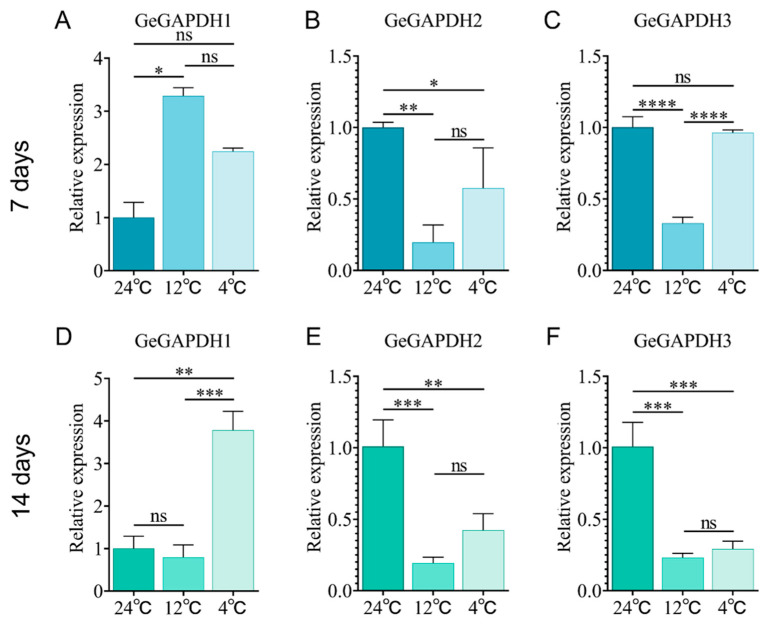
Relative expression of *GeGAPDH* family in *G. elata* under low-temperature stress. (**A**–**C**) Relative expression levels of *GeGAPDH* genes were treated at different temperatures on day 7. (**D**–**F**) Relative expression levels of *GeGAPDH* genes were treated at different temperatures on day 14. The X-axis represents different temperatures, and the Y-axis represents the relative expression levels normalized to the expression values of 24 °C. Error bars represent the standard error. Significant differences are indicated as follows: ns, non-significant; * *p* < 0.05; ** *p* < 0.01; *** *p* < 0.001; **** *p* < 0.0001. Values represent means ± SD (*n* = 4).

Temperature plays a crucial role in the quality and growth cycle of *G. elata*. To elucidate the potential role that *GeGAPDH* genes may play in responding to low-temperature stress, we exposed the tubers to different temperatures (24 °C, 12 °C, and 4 °C) and analyzed gene expression patterns of *GeGAPDH* genes. The results revealed distinct expression patterns of *GeGAPDH* genes under low-temperature stress. Compared to the optimal growth temperature of 24 °C, *GeGAPDH1* expression was significantly up-regulated on day 7 at 12 °C (Figure 11A) but returned to baseline levels on day 14 (Figure 11D). Additionally, *GeGAPDH1* expression remained stable on day 7 but showed a marked increase on day 14 at 4 °C (Figure 11A,D). In contrast, *GeGAPDH2* and *GeGAPDH3* showed distinct expression patterns under low-temperature stress conditions. At 12 °C, both genes were significantly downregulated at 7 and 14 days. Under 4 °C treatment, *GeGAPDH2* expression was consistently suppressed at both time points, whereas GeGAPDH3 displayed a delayed response—maintaining stable expression at day 7 but showing significant downregulation at day 14 (Figure 11B,C,E,F).

In conclusion, *GeGAPDH1* was up-regulated, whereas *GeGAPDH2* and *GeGAPDH3* were downregulated under low-temperature stress. These different expression profiles suggest that *GeGAPDH* subtypes may be regulated by different mechanisms and play different functional roles in response to low-temperature stress.

### 2.11. Expression Characteristics of the GeGAPDH Family Under Pathogen Stress

**Figure 12:**
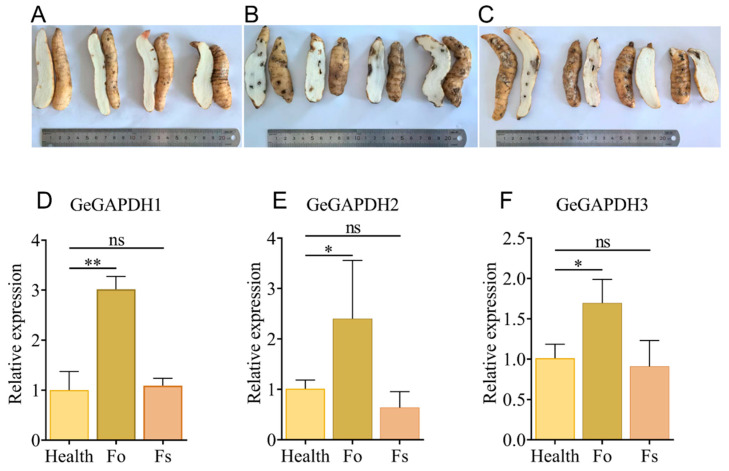
Relative expression of *GeGAPDH* family in *G. elata* under *Fusarium* stress. (**A**) Healthy control: *G. elata* treated with PDA medium for 7 days; (**B**,**C**) *G. elata* were infected by *F. oxysporum* (**B**) and *F. solani* (**C**) for 7 days, respectively, showing typical symptoms of brown rot. (**D**–**F**) Relative expression levels of *GeGAPDH* genes under pathogen stress for 7 days. Health represents the healthy control group, Fo represents the results of *F. oxysporum* infection, and Fs represents the results of *F. solani* infection. The X-axis represents different fungal pathogen treatments; the Y-axis represents relative expression levels normalized to the expression values of healthy *G. elata*. Error bars represent the standard error. Significant differences are indicated as follows: ns, non-significant; * *p* < 0.05; ** *p* < 0.01. Values represent means ± SD (*n* = 4).

During the cultivation of *G. elata*, the tubers are frequently susceptible to infections by pathogenic microorganisms. One prevalent type of infection is brown rot, caused by *Fusarium* spp. This study investigates the impact of two brown rot fungal pathogens, *F. oxysporum* and *F. solani*, on the tubers of *G. elata*. After a 7-day treatment with *F. oxysporum* and *F. solani*, the tubers showed characteristic symptoms of brown rot, including the appearance of dark brown lesions on the surface, which were sunken and soft with an unpleasant odor. Additionally, surrounding tissues developed concentric gray-brown lesions (Figure 12A–C). Gene expression analysis revealed that infection with *F. oxysporum* significantly up-regulated the expression of *GeGAPDH1*, *GeGAPDH2* and *GeGAPDH3*, with expression levels increasing to 3.02-, 2.56- and 1.69-fold, respectively, compared to healthy tubers. In contrast, infection with *F. solani* did not result in any detectable changes in the expression levels of *GeGAPDH* family members (Figure 12D–F). These results suggest that the *GeGAPDH* genes may play regulatory roles in pathogen stress responses, providing insights for further functional studies.

### 2.12. Detection of GAPDH Enzyme Activity Under Low Temperature and Fusarium Stress

**Figure 13:**
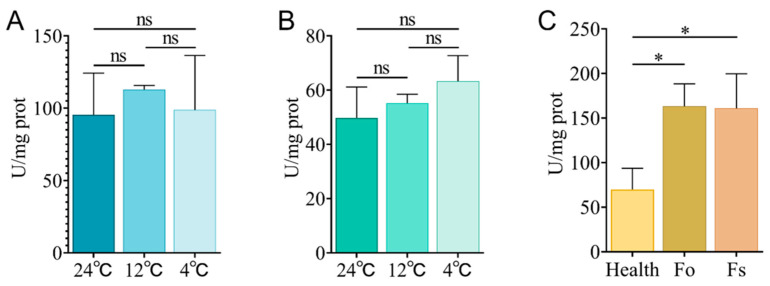
GAPDH enzyme activity under low temperature and *Fusarium* stress. (**A**) The results of GAPDH enzyme activity assay under low-temperature stress for 7 days. (**B**) The results of GAPDH enzyme activity assay under low-temperature stress for 14 days. (**C**) The results of GAPDH enzyme activity assay exposed to *Fusarium* stress for 7 days. Health represents the healthy control group, Fo represents the results of *F. oxysporum* infection, and Fs represents the results of *F. solani* infection. Error bars represent the standard error. Significant differences are indicated as follows: ns, non-significant; * *p* < 0.05. Values represent means ± SD (*n* = 4).

To further characterize *GeGAPDH* responses to stress conditions, total GAPDH enzyme activity was measured in tubers subjected to different stress conditions. Under low-temperature stress, the total GAPDH enzyme activity in *G. elata* tubers showed no significant alteration (Figure 13A,B). However, in pathogen infection assays, both *F*. *oxysporum* and *F*. *solani* infections significantly increased GAPDH enzyme activity (Figure 13C).

## 3. Discussion

Glyceraldehyde-3-phosphate dehydrogenase (GAPDH) not only plays a crucial role in the glycolytic and gluconeogenic pathways within plants but also exhibits diverse biological functions. These include mediating plant responses to abiotic stresses such as drought [40], high temperature [41], low temperature [42], and salinity [29,30], as well as involvement in hormone signaling [32] and processes related to viral infection [43] and immune response [13]. In this study, three *GAPDH* genes were identified, and physicochemical property predictions suggest that the proteins encoded by these genes are all basic hydrophilic proteins. Conserved motif analysis of the amino acid sequences indicates that motif3 of GeGAPDH contains the Gp_dh_N domain (INGFGRIGR), while motif4 contains the Gp_dh_C domain (GAAKAV) (Figure 1). Phylogenetic analysis classified GAPDH proteins into four subgroups, corresponding to distinct subtypes, and suggested that GeGAPDH2 and GeGAPDH3 were classified as cytosolic subtypes, potentially participating in the glycolytic process [44], whereas GeGAPDH1 clustered with the plastid subtype (Figure 2). This classification was supported by *N. benthamiana* transient transformation experiments (Figure 9), where GeGAPDH1-EGFP showed strong colocalization with chloroplasts. This distribution pattern is consistent with the distribution observed for *A. thaliana*, plastid GAPCp1 and GAPCp2 in the leaves [45]. As a non-photosynthetic plant, *G. elata* contains only non-green plastids and lacks chloroplasts, suggesting that GeGAPDH1 may function as a non-green plastid protein, providing important clues for predicting its role in *G elata*. Studies have found that although the GAPCp subtype does not play a major role in photosynthetic cells, it plays an important role in non-photosynthetic cells. They not only provide essential metabolites for plant survival but also affect nitrogen and carbon metabolism and mineral nutrition. Additionally, GAPCps are important for primary root growth and may be closely related to energy production during glycolysis [16,44,45,46]. For example, in *A. thaliana*, double mutants of the plastid subtypes GAPCp1 and GAPCp2 exhibit severe phenotypes, including root development impairment, dwarfism, and infertility. Supplementing growth media with serine can restore root development and normalize carbohydrate and sugar biosynthesis activity, indicating the critical role of GAPCps in serine synthesis in roots [45].

The biosynthesis of the primary active constituents of *G. elata*, including gastrodin, parishins, and polysaccharides, is closely related to glycosylation. The synthetic pathway typically utilizes UDP-glucose, derived from glucose, as a sugar donor, leading to the formation of polysaccharides or glycosides through glycosylation modifications [47,48]. Monosaccharides serve as the starting materials for this synthetic route and are critical for the accumulation of active components, with their biosynthesis primarily occurring via gluconeogenesis and the Calvin-Benson cycle [47]. The GAPDH mediates the reversible conversion between BPGA/NADH and G3P/inorganic phosphate/NAD^+^. In this study, the vitro enzymatic assays demonstrated that purified GeGAPDH1 from *G. elata* catalyzed BPGA dephosphorylation and G3P phosphorylation (Figure 7 and Figure 8). This activity mirrors the activities of both *GAPA/B* and *GAPCp* subtypes found in green plants [38], suggesting it may encompass the biological functions of both plant GAPA/B and GAPCp subtypes.

Tissue-specific expression analyses reveal that *GeGAPDH* is predominantly expressed in the medicinal tubers, followed by the stem and flowers (Figure 10). Notably, the tuber-predominant expression of *GeGAPDH* genes aligns with its potential role in medicinal compound biosynthesis, given that tubers are the primary site for bioactive metabolite accumulation.

Analysis of the cis-acting elements in the promoter region elucidates the regulatory mechanisms of genes and their tissue-specific expression patterns. Extensive studies have confirmed that *GAPDH* genes play significant roles in plant growth and development as well as in response to abiotic stresses. For instance, loss of cytosolic GAPC activity in *A. thaliana* leads to carbon flux disturbances and mitochondrial dysfunction, ultimately inhibiting plant growth [49]. Overexpression of *ClGAPC2* in watermelon and *GmGAPDH14* in soybean significantly enhances salt tolerance [29,30]. Moreover, *PlGAPC2* in *Herbaceous Peony* improves thermotolerance by inhibiting reactive oxygen species generation [50]. *GAPDH* genes in wheat and maize are implicated in drought response [20,25]. Notably, TaGAPCp1 protein localized in tobacco protoplasts showed significantly altered expression levels under osmotic stress and ABA induction [31]. GAPDH response to hormones and stress may be achieved through protein-protein interactions. TaGAPCp1 and its interacting protein cytochrome b6-f complex iron sulfite subunit (Cyt b6f) are involved in the response of wheat to abiotic stress, and this process may be completed by H_2_O_2_-mediated ABA signaling pathway [31]. Similarly, TaGAPC2 may interact with TaPLDδ to promote ABA-regulated stomatal closure, thereby enhancing drought tolerance in *T. aestivum* [40]. Additionally, *A. thaliana GAPCps* participates in abscisic acid signaling, influencing seed germination and plant growth [32]. Analysis of *GeGAPDH* gene promoters revealed various elements related to hormone responses, environmental stress, and light signaling (Figure 4). This suggests that the expression of the *GeGAPDH* genes may be regulated by multiple environmental and physiological conditions, thereby participating in plant growth and development as well as responses to abiotic stress. The findings of this study provide a theoretical basis for further exploring the potential functions of the *GeGAPDH* genes in regulating stress responses in *G. elata*.

The quality of *G. elata* is significantly related to the environmental temperature during its growth and storage [9]. Previous studies identified the glycolytic pathway as a primary pathway containing differentially expressed genes in response to low-temperature stress [9,51]. Metabolomics analyses reveal that *G. elata* grown at suitable temperatures contains significantly higher carbohydrate content compared to plants under low-temperature stress, where enzyme activity is inhibited, and carbohydrate metabolism is reduced, enhancing cold tolerance [9]. In this study, the expression of *GeGAPDH1* was up-regulated (Figure 11A,D), while *GeGAPDH2* and *GeGAPDH3* were down-regulated (Figure 11B,C,E,F) under low-temperature stress. Although the expression of the two types of genes in *G. elata* tubers showed different responses to low-temperature stress, the total GAPDH enzyme activity showed no significant change under different temperature stresses (Figure 13A,B). We propose two explanations: (1) there may be a compensatory mechanism such as post-translational modification in *GeGAPDH*; (2) The enzyme activity homeostasis may be due to the dynamic equilibrium between the two subtypes of *GeGAPDH* in the enzyme activity assay reaction.

*GAPDH* genes also play a crucial role in plant responses to pathogenic infections. The sensitivity of *A. thaliana AtGAPA*, *AtGAPC*, and *AtGAPCps* [13], cassava *MeGAPCs* [33], and *B. napus BnaGAPDH17*, *BnaGAPDH20*, *BnaGAPDH21*, and *BnaGAPDH22* [34] to pathogen infection have been demonstrated. *G. elata* is susceptible to pathogen infections during cultivation. This study analyzed *GeGAPDH* expression in healthy and brown rot pathogen-infected *G. elata*. The results revealed pathogen-specific variations in both GAPDH enzyme activity and gene expression patterns. After *F. oxysporum*-induced brown rot infection, all three *GeGAPDH* genes showed significant upregulation (Figure 12D–F), accompanied by markedly increased total GAPDH enzyme activity in *G. elata* tubers (Figure 13C). However, the *GeGAPDH* family members showed no significant transcriptional changes in *F. solani*-infected *G. elata* (Figure 12D–F) despite significant upregulation of total GAPDH enzyme activity (Figure 13C). The results of different expression levels showed that the *GeGAPDH* gene family may show pathogen-specific regulatory patterns. In response to *F. oxysporum* stress, the *GeGAPDH* gene family may directly participate in the regulation of disease resistance through the up-regulation of transcription levels. However, the increase in GAPDH enzyme activity during *F. solani* infection may depend on non-transcriptional regulatory mechanisms such as compensatory activation of other isoenzymes or post-translational modifications. These pathogen-specific regulatory strategies may reflect *G. elata*’s differential metabolic adaptation to distinct pathogen infection modes.

## 4. Materials and Methods

### 4.1. Identification of the GeGAPDH Family in G. elata

To identify the members of the *GAPDH* family in *G. elata*, the genome was downloaded from the National Genomics Science Data Center (accession number: GWHBHOU00000000) [52]. Initially, two hidden Markov models associated with conserved domains, Gp_dh_N (PF00044) and Gp_dh_C (PF02800), were obtained from the Pfam database [53]. Subsequently, using default parameters, hmmsearch (v.3.3.2) was employed to align the protein sequences of all genes in the *G. elata* genome with these two hidden Markov models. Genes that contained both conserved domains were preliminarily identified as candidate members of the *GAPDH* family. Next, the protein sequences of *A. thaliana GAPDH* genes were downloaded from the Phytozome v13 (https://phytozome-next.jgi.doe.gov/, accessed on 20 November 2024) [54]. BLASTP software (v.2.14.0) [55] was then utilized to compare the protein sequences of all genes in the *G. elata* genome against the *A. thaliana* GAPDH sequences, with an e-value ≤ 1 × 10^−5^. Ultimately, genes exhibiting both conserved domains and sequence homology were confirmed as members of the *GAPDH* family in *G. elata.*

### 4.2. Characterization of the GeGAPDH Family

All bioinformatics analyses were conducted using their respective default parameters as recommended by the tools. The physicochemical properties of the proteins were predicted using ProtParam (https://web.expasy.org/protparam/, accessed on 22 November 2024) [56]. The ProtScale tool (https://web.expasy.org/protscale/, accessed on 22 November 2024) [56] was employed to predict the hydrophilicity/hydrophobicity of the GeGAPDH proteins. The subcellular localization prediction was performed using WoLF PSORT (https://wolfpsort.hgc.jp/, accessed on 22 November 2024) [57]. Using TBtools (v2.096), the upstream 2000 bp sequences of the *GeGAPDH* genes were extracted based on the genomic annotation file. And PlantCARE (https://bioinformatics.psb.ugent.be/webtools/plantcare/html/, accessed on 23 November 2024) [58] was utilized to predict the cis-regulatory elements of *GeGAPDH*. Additionally, TBtools (v2.096) were used to analyze the structure and chromosomal localization of the *GeGAPDH* genes [59].

### 4.3. Collinearity, Phylogenetic and Protein–Protein Interaction Network Analysis of GAPDH Family

Protein sequences of GAPDH genes from *A. thaliana* and *O. sativa* were downloaded from the Phytozome database [54]. The One Step MCscanX module in TBtools (v2.096) was employed for pairwise comparison of the GAPDH protein sequences between *A. thaliana* and *G. elata*, as well as between *G. elata* and *O. sativa*. The Multiple Synteny Plot module was used to visualize the comparison results. *A. thaliana* GAPDH sequences were downloaded from the Phytozome v13 (https://phytozome-next.jgi.doe.gov/, accessed on 20 November 2024) [54], and *C. sinensis* and *T. aestivum* GAPDH sequences were obtained from the literature [20,37]. Next, Phylogenetic analysis was performed by TBtools (v2.096) “One Step Build a ML Tree” plugin with a bootstrap value set at 1000. Finally, the resulting phylogenetic tree was visualized and annotated using ChiPlo (https://www.chiplot.online/, accessed on 8 April 2025) [60]. Protein-protein interaction networks for GeGAPDH proteins were predicted using the STRING database v12.0 [61]. The analysis was performed by first specifying *G. elata* as the target organism through the “add organism” function. The amino acid sequences of GeGAPDH1, GeGAPDH2 and GeGAPDH3 were then input as query proteins. The interaction score threshold was set to “highest confidence” (0.900), with a maximum of 50 interactors to display (first shell). The PPI network was constructed using the default “full STRING network” mode.

### 4.4. Detection of GAPDH Activity in Total Protein Extracted from G. elata

Total protein from *G. elata* was extracted using a plant protein extraction kit (Solarbio, Beijing, China) according to the manufacturer’s protocols. The total protein concentration was determined using the Bradford protein concentration assay kit (Beyotime, Shanghai, China) [62]. The activity of glyceraldehyde-3-phosphate dehydrogenase (GAPDH) in the total protein extract was measured using a GAPDH activity detection kit (Solarbio, Beijing, China). One unit of enzyme activity was defined as the consumption of 1 nmol of NADH per minute.

### 4.5. Cloning, Expression, and Subcellular Localization of the GeGAPDH Genes

#### 4.5.1. RNA Extraction and cDNA Synthesis

Samples of *G. elata* were collected from Zhaotong, Yunnan. The samples were cleaned and stored at −80 °C. Total RNA was extracted from the tubers, stems, and flowers of *G. elata* using the RNAprep Pure Plant Plus Kit (Tiangen Biotech, Beijing, China) according to the manufacturer’s instructions and subsequently reverse-transcribed into cDNA using the Hifair^®^ II 1st Strand cDNA Synthesis SuperMix for qPCR (Yeasen Biotech, Shanghai, China).

#### 4.5.2. Cloning of the *GeGAPDH* Genes

Using the predicted full-length CDs as a reference sequence, specific primers for GeGAPDH were designed using the Biorun website (https://www.biorun.com) (Table S2). PCR amplification was performed using cDNA from the tubers as the template with Taq PCR Master Mix (Sangon Biotech, Shanghai, China). DNA fragments were purified using a magnetic bead-based DNA gel recovery kit (Beyotime, Shanghai, China). The purified DNA fragments were then ligated into the M5 HiPer pTOPO-Blunt cloning kit (Mei5bio, Beijing, China) and transformed into E. coli DH5α competent cells. Following PCR amplification and agarose gel electrophoresis, products consistent with the target gene fragment size were selected for Sanger sequencing, and plasmids were extracted using a plasmid extraction kit (Beyotime, Shanghai, China).

#### 4.5.3. Expression, Purification, Enzyme Activity and Kinetics Detection of the *GeGAPDH* Genes

The expression vector pGEX-4T-1 was linearized using restriction enzymes Xho I and BamH I. Recombinant primers containing homologous sequences to the vector were designed using the CE Design primer design software available on the Vazyme website (http://www.vazyme.com) (Table S2). Next, PCR amplification was conducted using the plasmid containing the target gene as a template, employing TransStart^®^ FastPfu DNA Polymerase (TransGen Biotech, Beijing, China) to obtain the target gene fragment. The linearized vector was then ligated with the target fragment using the ClonExpress^®^ II One Step Cloning Kit (Vazyme, Nanjing, China). The ligation product was transformed into *E. coli* BL21 competent cells. Positive clones were confirmed through PCR and 1% agarose gel electrophoresis, followed by Sanger sequencing. Positive clones were inoculated into LB medium containing 100 μg/mL ampicillin and cultured at 37 °C, 220 rpm until the OD600 reached 0.5 to 0.7. IPTG was then added to a final concentration of 0.5 mmol/L, and induction was performed at 16 °C, 120 rpm for 16 h.

Proteins were purified using a GST-tagged protein purification kit (Beyotime, Shanghai, China). SDS-PAGE was applied to determine the size of the expressed protein bands, and the gel was stained and destained using a Coomassie Brilliant Blue staining kit (Beyotime, Shanghai, China). Images of the gel were captured using a multifunctional luminescent imaging system. The concentration of the purified protein was measured using the Bradford method. The GAPDH enzyme activity in the purified protein was assessed using a GAPDH activity detection kit (Solarbio, Beijing, China), with the purified protein from the empty vector pGEX-4T-1 serving as a control.

To investigate whether GeGAPDH1 catalyzes reversible reactions between G3P and BPGA with concomitant NADH oxidation/reduction, we performed bidirectional enzyme activity assays using purified GeGAPDH1, with GST protein serving as the negative control. All reactions were conducted in 200 μL volumes in 96-well plates at 25 °C, monitoring NADH absorbance at 340 nm every 10 s for 20 min using a Spark multimode microplate reader. For the dephosphorylation direction (BPGA → G3P), the reaction mixture contained 25 mM triethanolamine (Macklin, Shanghai, China), 0.2 mM ATP (Solarbio, Beijing, China), 1 U 3-Phosphoglyceric Phosphokinase (Shang-hai Yingxin, Shanghai, China), 0.1 mM NADH (Yeasen, Shanghai, China), and 2 mM BPGA (Aladdin, Shanghai, China), initiated by adding 10 μg of purified GeGAPDH1. The phosphorylation direction (G3P → BPGA) reaction system consisted 50 mM triethanolamine, 50 mM sodium bisphosphate (Sinopharm, Beijing, China), and 0.2 mM EDTA (Beyotime, Shanghai, China), 0.6 mM NAD^+^ (Yeasen, Shanghai, China), and 2 mM G3P (Shanghai yuanye, Shanghai, China), started with 2.5 μg GeGAPDH1.

Enzyme kinetic experiments were performed in the phosphorylation direction by varying G3P concentrations (0.25–5 mM) under otherwise identical conditions. Reaction velocities were determined by monitoring the absorbance increase at 340 nm during the initial 5 min. The K_m_ and V_max_ were determined by non-linear least square fitting of the curve using GraphPad Prism 10. Assays were performed in triplicate.

#### 4.5.4. Subcellular Localization of the *GeGAPDH1* Gene

Primers were designed based on the CDs of GeGAPDH1 (Table S2). The subcellular localization vector pCAMBIA1300-35S-EGFP was linearized using the restriction enzymes SalI-HF and BamHI-HF. Then, using the method described in 4.5.3, the target gene fragment was obtained by PCR, and the gene was cloned into the subcellular localization vector pCAMBIA1300-35S-EGFP to construct the fusion plasmid. The ligation product was transformed into Agrobacterium GV3101 competent cells. Positive clones were confirmed through PCR and 1% agarose gel electrophoresis, followed by Sanger sequencing. The confirmed positive bacterial solution was inoculated in LB liquid medium (containing 50 μg/mL Kanamycin and Rifampicin) and cultured overnight at 28 °C at 200 rpm. The bacterial solution was centrifuged at 8000 rpm for 5 min to collect bacterial precipitates. The bacterial pellet was resuspended in Agrobacterium infection solution (Coolaber, Beijing, China) and adjusted OD600 to about 0.6. The resuspended solution was injected into leaves of *N*. *benthamiana*, which were incubated at 25 °C for 36 h in the dark and then incubated at the same temperature for 8 h in the dark and 16 h in the light for an additional 7 days. *N. benthamiana* leaves injected with Agrobacterium carrying the empty vector served as negative controls. Subcellular localization was observed using a laser scanning confocal microscope (Leica TCS SP8 STED, Wetzlar, Germany) with the following parameters: GFP fluorescence was excited at 488 nm and detected at 500–550 nm; chloroplast autofluorescence was excited at 488 nm and collected at 650–750 nm.

### 4.6. Expression Profile Analysis of the GeGAPDH Family

*GeActin* was selected as the internal reference gene [63]. Given that the CDs regions of *GeGAPDH2* and *GeGAPDH3* differ by only a single nucleotide, we implemented the following primer design strategy to ensure specificity in qRT-PCR detection: First, gene-specific primers for *GeGAPDH2* and *GeGAPDH3* were designed by targeting both their CDs regions and non-homologous segments within the untranslated regions of their respective mRNAs. Second, primers for *GeGAPDH1* were designed based solely on its CD sequence. This approach effectively prevents potential quantification bias that might arise from differential regulation due to distinct promoter elements between the paralogs. Real-time quantitative PCR amplification was performed using the Hieff qPCR SYBR Green Master Mix (Yeasen Biotech, Shanghai, China) by a two-step method. The relative expression level of *GeGAPDH* genes was analyzed using the 2^−ΔΔCt^ method. Each sample contained three biological replicates versus three technical replicates, and the relative expression level was the mean of three technical replicates out of three biological replicates.

#### 4.6.1. Effect of Low-Temperature Stress on *GeGAPDH* Expression in *G. elata* in Symbiotic with *A. mellea*

*A. mellea* and *G. elata* were cultured in tissue culture flasks. The potato dextrose agar (PDA) medium was prepared by dissolving 400 g/L potato infusion powder, 20 g/L glucose, and 15 g/L agar in distilled water, followed by pressure steam sterilization for 20 min at 121 °C. The *A. millaria* was inoculated into a PDA medium and cultured at 24 °C in the dark until the rhizomorphs covered the medium. Then, basal medium (wheat bran: PDA liquid medium = 1:0.6) was added for further culture. *G. elata* was cleaned, sterilized with 75% ethanol, and inoculated with the *A. millaria.* Samples were taken after dark incubation at 24 °C, 12 °C, and 4 °C for 7 and 14 days. RNA and first-strand cDNA were obtained from these samples, and the relative expression levels of *GeGAPDH* genes in tubers of *G. elata* at different temperatures were analyzed. The experimental design incorporated four biological replicates per treatment group, with each biological sample analyzed in three technical replicates. The relative expression level was the mean of three technical replicates out of four biological replicates.

#### 4.6.2. Effect of *F. oxysporum* and *F. solani* Stress on *GeGAPDH* Expression in *G. elata* in Symbiotic with *A. mellea*

The *F. oxysporum* and *F. solani* strains used in this study were isolated in November 2022 from *Salvia miltiorrhiza* exhibiting characteristic leaf spot and root rot symptoms, collected from Miaoshan Town, Laiwu District, Shandong Province, China [64]. Following isolation, both strains were maintained in our laboratory through periodic subculturing on PDA medium at 28 °C. *F. oxysporum* or *F. solani* were individually cultured on PDA plates at 28 °C in the dark until the pathogen reached two-thirds of the diameter of the plate, and the pathogen was taken for further experiments. *G. elata* was cleaned and sterilized with 75% ethanol. Ten uniform punctures (1 mm in diameter) were made on each tuber using a sterile syringe. A 5 mm-diameter disc of *F. oxysporum* or *F. solani* was placed on the punctured sites. *G. elata* inoculated on PDA was used as a control. The treated samples were then inoculated with *A. millaria* as described above and incubated at 24 °C in the dark for 7 days before sampling. RNA and first-strand cDNA were obtained from these samples, and the relative expression levels of *GeGAPDH* genes in tubers of *G. elata* under pathogen stress were analyzed. The experimental design incorporated four biological replicates per treatment group, with each biological sample analyzed in three technical replicates. The relative expression level was the mean of three technical replicates out of four biological replicates.

#### 4.6.3. Detection of GAPDH Enzyme Activity Under Low Temperature and Pathogen Stress Conditions

The activity of the GAPDH enzyme was measured in stress-treated *G. elata* samples (as described in Section 4.6.2 and Section 4.6.3) using a GAPDH activity detection kit (Solarbio, Beijing, China). The experimental design incorporated four biological replicates per treatment group, with each biological sample analyzed in three technical replicates. The reported enzyme activity values were the mean of three technical replicates out of four biological replicates.

#### 4.6.4. Data Analysis

Statistical analyses were performed using Excel 2021 and GraphPad Prism 10. Group comparisons were made using one-way ANOVA, with statistical significance defined as *p* < 0.05.

## 5. Conclusions

This study systematically identified three *GeGAPDH* genes in *G. elata*, all encoding stable hydrophilic proteins. Phylogenetic analysis indicated that *GeGAPDH1* is a plastid subtype, while *GeGAPDH2* and *GeGAPDH3* are cytosolic subtypes. Heterologous expression confirmed that *GeGAPDH1* is localized in plastids and exhibits significant phosphorylating and dephosphorylating GAPDH enzyme activity. Expression analysis revealed predominant *GeGAPDH* expression in tubers. Furthermore, the expression levels of the different *GeGAPDH* subtypes in tubers are influenced by low-temperature and pathogen stresses, exhibiting differential expression patterns under various stress conditions. These findings elucidate the functional differentiation between *GeGAPDH* subtypes in heterotrophic plant *G. elata* and their distinct regulatory responses to environmental stress, laying the foundation for further research on *GAPDH* gene functions.

## Figures and Tables

**Table 1:** Physicochemical characterization and subcellular localization of the *GeGAPDH* family in *G. elata*.

Gene Name	Gene ID	Number of Amino Acids	Molecular Mass	Theoretical pI	Instability Index ^1^	Grand Average of Hydropathicity ^2^	Subcellular Prediction
*GeGAPDH1*	GWHGBHOU003488	428	45,867.01	8.51	37.51	−0.106	plastid
*GeGAPDH2*	GWHGBHOU007564	344	37,088.64	7.68	26.75	−0.01	cytoplasm
*GeGAPDH3*	GWHGBHOU007572	344	37,088.64	7.68	26.75	−0.01	cytoplasm

^1^ The instability index provides an estimate of the protein stability. A protein whose instability index is smaller than 40 is predicted as stable; a value above 40 predicts that the protein may be unstable; ^2^ The grand average of hydropathy value for a peptide or protein is calculated as the sum of hydropathy values [36] of all the amino acids, divided by the number of residues in the sequence.

## Data Availability

All data supporting this study are available within the paper and within the Supplementary Materials published online.

## Supplementary Materials

The following supporting information can be downloaded at: https://www.mdpi.com/article/10.3390/plants14121866/s1, Figure S1: Sequencing results of *GeGAPDH1* gene in cloningvector; Figure S2: Sequencing results of *GeGAPDH1* gene in expression vector; Figure S3: Sequencing results of *GeGAPDH1* gene in subcellular localization vector; Table S1: The results of homologous sequence alignment and HMM search; Table S2: The primer sequences used in this project; Data S1: The GAPDH protein sequences in phylogenetic tree.
