# Supplementary material for: Identification of the Glyceraldehyde-3-Phosphate Dehydrogenase (GeGAPDH) Gene Family in Gastrodia elata Revealing Its Response Characteristics to Low-Temperature and Pathogen Stress
Source: Plants (Basel). 2025 Jun 18;14(12):1866. doi: 10.3390/plants14121866 (PMC12197075; doi:10.3390/plants14121866)
Supplement: Supplementary file 1 [file plants-14-01866-s001.zip › plants-3495831-supplementary.pdf]

Supplementary Files

Title

Identification of the Glyceraldehyde-3-phosphate dehydrogenase (GeGAPDH) Gene Family in *Gastrodia elata* Revealing Its Response Characteristics to Low-temperature and Pathogen Stress

Yaxing Yan[1,2], Mei Jiang[3,4], Pengjie Han [3,4], Xiaohu Lin [1,2*] and Xiao Wang[3,4*]

Affiliations

1 College of Agronomy and Biotechnology, Hebei Key Laboratory of Crop Stress Biology, Hebei Normal University of Science and Technology, Qinhuangdao 066000, Hebei Province, China; 18149114906@163.com

2 Analysis and Testing Center, Hebei Normal University of Science and Technology, Qinhuangdao 066000, Hebei Province, China

3 Shandong Engineering Research Center for Innovation and Application of General Technology for Sepa-ration of Natural Products, Shandong Analysis and Test Center, Qilu University of Technology, Jinan 250014, Shandong Province, China; mjiang0502@163.com (M.J.); hanpengjie2023@126.com (P.H.)

4 Key Laboratory for Natural Active Pharmaceutical Constituents Research in Universities of Shandong Province, School of Pharmaceutical Sciences, Qilu University of Technology, Jinan 250014, Shandong Province, China

* Correspondence: xiaohulin2008@163.com (X.L.); wangx@sdas.org (X.W.)

Table S1. The results of homologous sequence alignment and HMM search.

ID	homologous sequence alignment									HMM search			
										full sequence		best 1 domain	
	query	subject	percentage	length	start in query	end in query	start in subject	end in subject	E-value	E-value	score	E-value	score
GeGAPDH1	AT1G79530.1	GWHPBHOU006545	83.607	366	55	420	61	426	0	1.40E-37	129	3.10E-37	127.9
GeGAPDH2	AT1G79530.1	GWHPBHOU014178	72.590	332	86	416	6	337	3.76e-178	6.60E-33	114.2	1.40E-32	113
GeGAPDH3	AT1G79530.1	GWHPBHOU014187	72.590	332	86	416	6	337	3.76e-178	6.60E-33	114	1.70E-32	112.7

Table S2. The primer sequences used in this project.

ID	Sequence	Function
GeGAPDH1_C_F	ATGGCTTCTACCGCTTTCGTA	Gene cloning
GeGAPDH1_C_R	TCATTTATGCGCACAGACACG	
GeGAPDH1_E_F	gatctggttccgcgtggatccATGGCTTCTACCGCTTTCGTAG	Gene expression
GeGAPDH1_E_R	gtcacgatgcggccgctcgagTCATTTATGCGCACAGACACG	
GeGAPDH1_L_F	cgagctcggtacccgggatccATGGCTTCTACCGCTTTCGTAG	Subcellular localization
GeGAPDH1_L_R	cgcgtacgagatctgggtcgacTTTATGCGCACAGACACGAGC	
GeGAPDH1-qpcr-F	GGACTTGACTTGCCGGCT	qRT-PCR
GeGAPDH1-qpcr -R	AAGCAACCCTCCGATGCC	
GeGAPDH2-qpcr -F	CGATTTATTTTCCTTTTTGAATCG	
GeGAPDH2-qpcr -R	CTCCACATCTTCGCTCAACAGC	
GeGAPDH3-qpcr -F	TGAGGACGCCAGATGAGATG	
GeGAPDH3-qpcr -R	ACTCGCCAACATCGACGAAC	
Actin-F	GCAGCATGAAGATCAAGGTGG	
Actin-R	GCCTTAGAAATCCACATCTGTTG	

The cutting site of the restriction enzymes are indicated in red font.

Data S1. The GAPDH protein sequences in phylogenetic tree.

>GeGAPDH1
MASTAFVGSVTPLSEASFPFASSTRAPEPFRVFCWRSRSSEGSSVLCASTSCSKFPLAAISTQKSNRRNFQHTKATATETPP
VVSRSSSDGKTKVGINGFGRIGRLVLRIAASRDDIEVVAVNDPFVDAKYMAYMFKYDSTHGTFKGTIKVVDESTIEIDGKRI
AVTSKRDPSEITWSDFGAEYVVESSGVFTTMDKASAHLKGGAKKVVISAPSADAPMFVVGVNEQMYQPHMNIVSNAS
CTTNCLALLAKVVHEEFGIVEGLMTTVHATTATQKTVDGPSMKDWRGGRGASQNIIPSSTGAAKAVGKVLPELNGKLTG
MAFRVPTTNVSVVDLTCRLANDASYEDVKAAVRAASEGCLKGILGYTDEDVVSNDFIGDSRSSIFDAKAGIGLGTNFMKL
VSWYDNEWGYSNRVLDLIQHVARVCAHK
>GeGAPDH2
MAPTRKIKLGINGFGRIGRLVARVALLSEDVELVAVNDPFITTDYMTYMFKYDTVHGQWKHHEITVKDSKTLLFGEKPVTV
FGVRNPEEIPWGEAGADFVVESTGVFTDKDKAAAHLKGGAKKVIISAPSKDAPMFVVGVNEKEYKADLDIVSNASCTTN
CLAPLAKVIHDKFGIIEGLMTTVHAITATQKTVDGPSSKDWRGGRAASFNIIPSSTGAAKAVGKVLPELNGKLTGMSLRVP
TVDVSVIDLTVRIEKKASYDQIKAAVKAESEGKLKGIIGYTDLDLVSSDFLGDSRSCIFDAKAGIALNDGFVKLVAWYDNEW
GYSNRVVDLIRHIAGTIAAAQ
>GeGAPDH3
MAPTRKIKLGINGFGRIGRLVARVALLSEDVELVAVNDPFITTDYMTYMFKYDTVHGQWKHHEITVKDSKTLLFGEKPVTV
FGVRNPEEIPWGEAGADFVVESTGVFTDKDKAAAHLKGGAKKVIISAPSKDAPMFVVGVNEKEYKADLDIVSNASCTTN
CLAPLAKVIHDKFGIIEGLMTTVHAITATQKTVDGPSSKDWRGGRAASFNIIPSSTGAAKAVGKVLPELNGKLTGMSLRVP
TVDVSVIDLTVRIEKKASYDQIKAAVKAESEGKLKGIIGYTDLDLVSSDFLGDSRSCIFDAKAGIALNDGFVKLVAWYDNEW
GYSNRVVDLIRHIAGTIAAAQ
>AtGAPA1
MASVTFSVPKGFTEFSGLRSSSASLPFGKKLSSDEFVSIVSFQTSAMGSSGGYRKGVTEAKLKVAINGFGRIGRNFLRCWH
GRKDSPLDIIAINDTGGVKQASHLLKYDSTLGIFDADVKPSGETAISVDGKIIQVVSNRNPSLLPWKELGIDIVIEGTGVFVD
REGAGKHIEAGAKKVIITAPGKGDIPTYVVGVNADAYSHDEPIISNASCTTNCLAPFVKVLDQKFGIIKGTMTTTHSYTGD
QRLLDASHRDLRRARAAALNIVPTSTGAAKAVALVLPNLKGKLNGIALRVPTPNVSVVDLVVQVSKKTFAEEVNAAFRDS
AEKELKGILDVCDEPLVSVDFRCSDFSTTIDSSLTMVMGDDMVKVIAWYDNEWGYSQRVVDLADIVANNWK

>AtGAPA2
MVVRTLLLMSLSLTTLVVLNKHHISSNTTQLLESLTLMSNLQETQLSLLMERSSRLYLIVTHLISPGGKELGIDLVIEGTGVFV
DRDGAGKHLQAGAKKVLITAPGKGDIPTYVVGVNAELYSHEDTIISNASCTTNCLAPFVKVLDQKFGIIKGTMTTTHSYTG
DQRLLDASHRDLRRARAAALNIVPTSTGAAKAVALVLPNLKGKLNGIALRVPTPNVSVVDLVVQVSKKTFAEEVNAAFR
DAAEKELKGILDVCDEPLVSVDFRCSDVSSTIDSSLTMVMGDDMVKVIAWYDNEWGYSQRVVDLADIVANNWK
>AtGAPB
MATHAALAVSRIPVTQRLQSKSAIHSFPAQCSSKRLEVAEFSGLRMSSIGGEASFFDAVAAQIIPKAVTTSTPVRGETVAKL
KVAINGFGRIGRNFLRCWHGRKDSPLEVVVLNDSGGVKNASHLLKYDSMLGTFKAEVKIVDNETISVDGKLIKVVSNRDP
LKLPWAELGIDIVIEGTGVFVDGPGAGKHIQAGASKVIITAPAKGADIPTYVMGVNEQDYGHDVANIISNASCTTNCLAP
FAKVLDEEFGIVKGTMTTTHSYTGDQRLLDASHRDLRRARAAALNIVPTSTGAAKAVSLVLPQLKGKLNGIALRVPTPNV
SVVDLVINVEKKGLTAEDVNEAFRKAANGPMKGILDVCDAPLVSVDFRCSDVSTTIDSSLTMVMGDDMVKVVAWYDN
EWGYSQRVVDLAHLVASKWPGAEAVGSGDPLEDFCKTNPADEECKVYD
>AtGAPC1
MADKKIRIGINGFGRIGRLVARVVLQRDDVELVAVNDPFITTEYMTYMFKYDSVHGQWKHNELKIKDEKTLLFGEKPVTV
FGIRNPEDIPWAEAGADYVVESTGVFTDKDKAAAHLKGGAKKVVISAPSKDAPMFVVGVNEHEYKSDLDIVSNASCTTN
CLAPLAKVINDRFGIVEGLMTTVHSITATQKTVDGPSMKDWRGGRAASFNIIPSSTGAAKAVGKVLPALNGKLTGMSFR
VPTVDVSVVDLTVRLEKAATYDEIKKAIKEESEGKLKGILGYTEDDVVSTDFVGDNRSSIFDAKAGIALSDKFVKLVSWYDN
EWGYSSRVVDLIVHMSKA
>AtGAPC2
MADKKIRIGINGFGRIGRLVARVVLQRDDVELVAVNDPFITTEYMTYMFKYDSVHGQWKHHELKVKDDKTLLFGEKPVT
VFGIRNPEDIPWGEAGADFVVESTGVFTDKDKAAAHLKGGAKKVVISAPSKDAPMFVVGVNEHEYKSDLDIVSNASCTT
NCLAPLAKVINDRFGIVEGLMTTVHSITATQKTVDGPSMKDWRGGRAASFNIIPSSTGAAKAVGKVLPSLNGKLTGMSF
RVPTVDVSVVDLTVRLEKAATYDEIKKAIKEESEGKMKGILGYTEDDVVSTDFVGDNRSSIFDAKAGIALSDKFVKLVSWY
DNEWGYSSRVVDLIVHMSKA
>AtGAPCp1
MAFSSLLRSAASYTVAAPRPDFFSSPASDHSKVLSSLGFSRNLKPSRFSSGISSSLQNGNARSVQPIKATATEVPSAVRRSS
SSGKTKVGINGFGRIGRLVLRIATSRDDIEVVAVNDPFIDAKYMAYMLKYDSTHGNFKGSINVIDDSTLEINGKKVNVVSK
RDPSEIPWADLGADYVVESSGVFTTLSKAASHLKGGAKKVIISAPSADAPMFVVGVNEHTYQPNMDIVSNASCTTNCLA
PLAKVVHEEFGILEGLMTTVHATTATQKTVDGPSMKDWRGGRGASQNIIPSSTGAAKAVGKVLPELNGKLTGMAFRVPT
SNVSVVDLTCRLEKGASYEDVKAAIKHASEGPLKGILGYTDEDVVSNDFVGDSRSSIFDANAGIGLSKSFVKLVSWYDNE
WGYSNRVLDLIEHMALVAASH
>AtGAPCp2
MALSSLLRSAATSAAAPRVELYPSSSYNHSQVTSSLGFSHSLTSSRFSGAAVSTGKYNAKRVQPIKATATEAPPAVHRSRS
SGKTKVGINGFGRIGRLVLRIATFRDDIEVVAVNDPFIDAKYMAYMFKYDSTHGNYKGTINVIDDSTLEINGKQVKVVSKR
DPAEIPWADLGAEYVVESSGVFTTVGQASSHLKGGAKKVIISAPSADAPMFVVGVNEKTYLPNMDIVSNASCTTNCLAP
LAKVVHEEFGILEGLMTTVHATTATQKTVDGPSMKDWRGGRGASQNIIPSSTGAAKAVGKVLPELNGKLTGMAFRVPTP
NVSVVDLTCRLEKDASYEDVKAAIKFASEGPLRGILGYTEEDVVSNDFLGDSRSSIFDANAGIGLSKSFMKLVSWYDNEW
GYSNRVLDLIEHMALVAASR
>TaGAPDH1
MASPMLSTAMAPLQGGMLEFSGLRSSSSLPLRRNATSDDFMSAVSFRTYAAAALCLQVSTSGGSRKAPTEAKLKVAING
FGRIGRNFLRCWHGRGDSSPLEVIAINDTGGVKQASHLLKYDSTLGIFDADVKPVGDNAISVDGKVIKVVSDRNPSNLP
WGEMGIDLVIEGTGVFVDRAGAGKHLEAGAKKVLITAPGKGDIPTYVCGVNADLYTHADTIISNASCTTNCLAPFVKVLD
QKFGIIKGTMTTTHSYTGDQRLLDASHRDLRRARAAALNIVPTSTGAAKAVALVLPNLKGKLNGIALRVPTPNVSVVDLV
VQVSKKTLAEEVNQAFRDAAANELKGILDVCDEPLVSVDFRCSDVSSTIDASLSMVMGDDMVKVIAWYDNEWGYSQR

VVDLADIVADQWK
>TaGAPDH2
MASPMLSTAMAPLQGGMLEFSGLRSSSSLPLRRNATSDDFMSAVSFRTYAAAVLCLQVSTSGGSRKAPTEAKLKVAING
FGRIGRNFLRCWHGRGDSSPLEVIAINDTGGVKQASHLLKYDSTLGIFDADVKPVGDNAISVDGKVIKVVSDRNPSNLP
WGEMGIDLVIEGTGVFVDRAGAGKHLEAGAKKVLITAPGKGDIPTYVCGVNADLYTHADTIISNASCTTNCLAPFVKVLD
QKFGIIKGTMTTTHSYTGDQRLLDASHRDLRRARAAALNIVPTSTGAAKAVALVLPNLKGKLNGIALRVPTPNVSVVDLV
VQVSKKTLAEEVNQAFRDAAANELKGILDVCDEPLVSVDFRCSDVSSTIDASLSMVMGDDMVKVIAWYDNEWGYSQR
VVDLADIVANQWK
>TaGAPDH3
MDGFLLCAAGFGRIGRLVARVALQSPDVELVAVNDPFITTDYMTYMFKYDTVHGQWKHHEVKVKDSKTLLFGQKEVAV
FGCRNPEEIPWAAAGAEYVVESTGVFTDKDKAAAHIKGGAKKVIISAPSKDAPMFVCGVNEKEYKSDIDIVSNASCTTNC
LAPLAKVINDRFGIVEGLMTTVHAMTATQKTVDGPSSKDWRGGRAASFNIIPSSTGAAKAVGKVLPELNGKLTGMAFRV
PTVDVSVVDLTVRLAKPATYDQIKAAIKEESEGNLKGILGYVDEDLVSTDFQGDNRSSIFDAKAGIALNDNFVKLVSWYD
NEWGYSTRVVDLIRHMHSSK
>TaGAPDH4
MAPIKIGINGFGRIGRLVARVALQSPDVELVAVNDPFITTDYMTYMFKYDTVHGQWKHHEVKVKDSKTLLFGQKEVAVF
GCRNPEEIPWAAAGAEYVVESTGVFTDKDKAAAHIKGGAKKVIISAPSKDAPMFVCGVNEKEYKSDIDIVSNASCTTNCL
APLAKVINDRFGIVEGLMTTVHAMTATQKTVDGPSSKDWRGGRAASFNIIPSSTGAAKAVGKVLPELNGKLTGMAFRVP
TVDVSVVDLTVRLAKPATYDQIKAAIKEESEGNLKGILGYVDEDLVSTDFQGDNRSSIFDAKAGIALNDNFVKLVSWYDN
EWGYSTRVVDLIRHMHSSK
>TaGAPDH5
AGSRAAADPSKVSCVRSTRSAHFDCSFPSIAASSSSARNIEPLRAIATQAPPAVPQYLSGEKTKVGINGFGRIGRLVLRIATS
RDDIEVVAVNDPFIDAKYMAYMFKYDSTHGPFKGSINVVDDSTLEINGKKITITSKRDPAEIPWGNFGADYVVESSGVFTT
IDKASVHLKGGAKKVVISAPSADAPMFVVGVNEMSYDPKMNVVSNASCTTNCLAPLAKVVHEEFGILEGLMTTVHATT
ATQKTVDGPSMKDWRGGRGAGQNIIPSSTGAAKAVGKVLPELNGKLTGMAFRVPTPNVSVVDLTCRLEKSASYDDVK
AAIKAASEGALKGILGYTDEDVVSNDFVGDTRSSVFDANAGMGLSSSFMKLVSWYDNEWGYSNRVLDLIAHMALVSAK
H
>TaGAPDH6
MKPRSLPVKFCINHPVCLVLLMAPIKIGINGFGRIGRLVARVALQSPDVELVAVNDPFITTDYMTYMFKYDTVHGQWKH
HEVKVKDSKTLLFGEKEVAVFGCRNPEEIPWAAAGAEYVVESTGVFTDKDKAAAHIKGGAKKVIISAPSKDAPMFVCGVN
EKEYKSDIDIVSNASCTTNCLAPLAKVINDRFGIVEGLMTTVHAMTATQKTVDGPSSKDWRGGRAASFNIIPSSTGAAKA
VGKVLPELNGKLTGMAFRVPTVDVSVVDLTVRLAKPATYDQIKAAIKEESEGNLKGILGYVDEDLVSTDFQGDNRSSIFDA
KAGIALNDNFVKLVSWYDNEWGYSTRVVDLIRHMHSTK
>TaGAPDH7
MPVSVAVALLLSTFVWNLLLCYYIASCVRSTGSAHFGCSFPSIVASSSSVRNIEPLRAIATQAPPAVPQYSSGEKTKVGING
FGRIGRLVLRIAISRDDIEVVAVNDPFIDAKYMAYMFKYDSTHGPFKGSINVVDDSTLEINGKKITITSKRDPAEIPWGNFGV
DYVVESSGVFTTIDKASVHLKGGAKKVVISAPSADAPMFVVGVNEMSYDPKMNVVSNASCTTNCLAPLAKVVHEEFGIL
EGLMTTVHATTATQKTVDGPSMKDWRGGRGAGQNIIPSSTGAAKAVGKVLPELNGKLTGMAFRVPTPNVSVVDLTCR
LEKSASYDDVKAAIKAASEGALEGILGYTDEDVVSNDFVGDTRSSIFDANAGMGLSSSFMKLVSWYDNEWGYSNRVLDL
IAHMALVTAKH
>TaGAPDH8
MAPIKIGINGFGRIGRLVARVALQSPDVELVAVNDPFITTDYMTYMFKYDTVHGQWKHHEVKVKDSKTLLFGEKEVAVF
GCRNPEEIPWAAAGAEYVVESTGVFTDKDKAAAHIKGGAKKVIISAPSKDAPMFVCGVNEKEYKSDIDIVSNASCTTNCL
APLAKVINDRFGIVEGLMTTVHAMTATQKTVDGPSSKDWRGGRAASFNIIPSSTGAAKAVGKVLPELNGKLTGMAFRVP

TVDVSVVDLTVRLAKPATYDQIKAAIKEESEGNLKGILGYVDEDLVSTDFQGDNRSSIFDAKAGIALNDNFVKLVSWYDN
EWGYSTRVVDLIRHMHSTK
>TaGAPDH9
MASLSLSLRASASSAAAGSRAAAPIKASCVRSKVTCSLPSIRATSSPARSIEPVRATATQAPPATPQSSSREKTKVGINGFG
RIGRLVLRIAIDRDDIEVVAVNDPFIDAKYMAYMFKYDSTHGPFKGTITVLDESTLEINGKKVSVTSKRDPSDIPWGNFGAE
YVVESSGVFTTVEKASAHLKGGAKKVVISAPSADAPMFVVGVNEKNYNPSMDVVSNASCTTNCLAPVAKVVHEEFGILE
GLMTTVHATTATQKTVDGPSMKDWRGGRGAGQNIIPSSTGAAKAVGKVLPALNGKLTGMAFRVPTPNVSVVDLTCRL
EKNASYEDVKAAIKEASEGSLKGILGYTDEDVVSNDFVGDTRSSIFDANAGMGLSSSFMKLVSWYDNEWGYSNRVLDLI
GHMSLVA
>TaGAPDH10
MGKIKIGINGFGRIGRLVARVALQSDDVELVAVNDPFITTEYMTYMFKYDTVHGHWKHSDIKLKDDKTLLFGEKPVTVFG
VRNPEEIPWGEAGADYVVESTGVFTDKDKAAAHLKGGAKKVVISAPSKDAPMFVVGVNEDKYTSDVNIVSNASCTTNC
LAPLAKIINDNFGIIEGLMTTVHAITATQKTVDGPSSKDWRGGRAASFNIIPSSTGAAKAVGKVLPELNGKLTGMSFRVPT
VDVSVVDLTVRTEKAASYDDIKKCLPAHVTRMSLMSIQIIFRAASEGKLKGIMGYVEEDLVSTDFVGDSRSSIFDAKAGIAL
NDHFVKLVSWYDNEWGYSNRVVDLIRHMAKTQ
>TaGAPDH11
ASLSLSLRANASASPAAAGSRAAASIKASCVRSKVTFSLPSIRATSSPARSIEPVRATATQAPPATPQTSSGEKTKVGINGF
GRIGRLVLRIAIDRDDIEVVAVNDPFIDAKYMAYMFKYDSTHGPFKGTITVLDESTLEINGKKVSVTSKRDPSDIPWGNFGA
EYVVESSGVFTTVEKASAHLKGGAKKVVISAPSADAPMFVVGVNEKNYNPSMDVVSNASCTTNCLAPVAKVVHEEFGIL
EGLMTTVHATTATQKTVDGPSMKDWRGGRGAGQNIIPSSTGAAKAVGKVLPALNGKLTGMAFRVPTPNVSVVDLTCR
LEKSASYEDVKAAIKEASEGSLKGILGYTDEDVVSNDFVGDTRSSIFDANAGMGLSSSFMKLVSWYDNEWGYSNRVLDLI
GHMALVNA
>TaGAPDH12
MGKIKIGINGFGRIGRLVARVALQSDDVELVAVNDPFITTEYMTYMFKYDTVHGHWKHSDIKLKDDKTLLFGEKPVTVFG
VRNPEEIPWGEAGADYVVESTGVFTDKDKAAAHLKGGAKKVVISAPSKDAPMFVVGVNEDKYTSDVNIVSNASCTTNC
LAPLAKIINDNFGIIEGLMTTVHAITATQKTVDGPSSKDWRGGRAASFNIIPSSTGAAKAVGKVLPELNGKLTGMSFRVPT
VDVSVVDLTVRTEKAASYDDIKKAIKAASEGKLKGIMGYVEEDLVSTDFVGDSRSSIFDAKAGIALNDHFVKLVSWYDNE
WGYSNRVVDLIRHMAKTQ
>TaGAPDH13
MASLSLSLRASASPAASGSRAAAPIKASCVRSKVACSFPSVGATSSPARSIEPVRATATQAPPASSQSSSGEKTKVGINGF
GRIGRLVLRIAIDRDDIEVVAVNDPFIDAKYMAYMFKYDSTHGPFKGTIAVLDESTLEINGKKVSVTSKRDPSDIPWGNFG
AEYVVESSGVFTTVEKASAHLKGGAKKVVISAPSADAPMFVVGVNEKNYNPSMDVVSNASCTTNCLAPVAKVVHEEFGI
LEGLMTTVHATTATQKTVDGPSMKDWRGGRGAGQNIIPSSTGAAKAVGKVLPALNGKLTGMAFRVPTPNVSVVDLTC
RLEKDASYEDVKAAIKEASEGPLKGILGYTDEDVVSNDFVGDTRSSIFDANAGMGLSSSFMKLVSWYDNEWGYSNRVLD
LIGHMVLVA
>AtGAPN
MAGTGLFAEILDGEVYKYYADGEWKTSSSGKSVAIMNPATRKTQYKVQACTQEEVNAVMELAKSAQKSWAKTPLWKR
AELLHKAAAILKDNKAPMAESLVKEIAKPAKDSVTEVVRSGDLISYCAEEGVRILGEGKFLLSDSFPGNDRTKYCLTSKIPLG
VVLAIPPFNYPVNLAVSKIAPALIAGNSLVLKPPTQGAVSCLHMVHCFHLAGFPKGLISCITGKGSEIGDFLTMHPAVNCIS
FTGGDTGISISKKAGMIPLQMELGGKDACIVLDDADLDLVASNIIKGGFSYSGQRCTAVKVVLVMESVADELVEKVKAKV
AKLTVGPPEENSDITAVVSESSANFIEGLVMDAKEKGATFCQEYKREGNLIWPLLLDNVRPDMRIAWEEPFGPVVPVLRIN
SVEEGINHCNASNFGLQGCVFTKDINKAILISDAMETGTVQINSAPARGPDHFPFQGLKDSGIGSQGVTNSINLMTKVKT
TVINLPTPSYSMG
>TaGAPN1

MAGTGVFADVLDGEVYKYYADGEWRASASGKTVAIVNPTTRQTQYRVQACTQEEVNKVMDAAKVAQKAWARTPLW
KRAELLHKAAAILKEHKTPIAECLVKEIAKPAKDAVSEVVRSGDLVSYTAEEGVRILGEGKLLVSDSFPGNERNKYCLSSKVP
LGVVLAIPPFNYPVNLAVSKIGPALIAGNSLVLKPPTQGAVAALHMVHCFHLAGFPKGLISCVTGKGSEIGDFLTMHPGV
NCISFTGGDTGIAISKKAGMVPLQMELGGKDACIVLEDADLDLVAANIVKGGFSYSGQRCTAVKVVLIMEAVADTVVEK
VNAKLAKLKVGPPEDDSDITPVVTESSANFIEGLVMDAKEKGATFCQEYRREGNLIWPLLLDHVRPDMRIAWEEPFGPVL
PVIRINSVEEGIHHCNASNFGLQGCVFTRDINKAIMISDAMESGTVQINSAPARGPDHFPFQGLKDSGIGSQGITNSINM
MTKVKSTVINLPSPSYTMG
>TaGAPN2
MAGTGVFADVLDGEVYKYYADGEWRASASGKTVAIVNPTTRQTQYRVQACTQEEVNKVMDAAKVAQKSWARTPLW
KRAELLHKAAAILKEHKTPIAECLVKEIAKPAKDAVSEVVRSGDLVSYTAEEGVRILGEGKLLVSDSFPGNERNKYCLSSKVP
LGVVLAIPPFNYPVNLAVSKIGPALIAGNSLVLKPPTQGAVAALHMVHCFHLAGFPKGLISCVTGKGSEIGDFLTMHPGV
NCISFTGGDTGIAISKKAGMVPLQMELGGKDACIVLEDADLDLVAANVVKGGFSYSGQRCTAVKVVLIMEAVADTVVEK
VNAKLAKLKVGPPEDDCDITPVVTESSANFIEGLVMDAKEKGATFCQEYRREGNLIWPLLLDHVRPDMRIAWEEPFGPVL
PVIRINSVEEGIHHCNASNFGLQGCVFTRDINKAIMISDAMESGTVQINSAPARGPDHFPFQGLKDSGIGSQGITNSINM
MTKVKSTVINLPSPSYTMG
>TaGAPN3
MDAAKVAQKAWARTPLWKRAELLHKAAAILKEHKAPIAECLVKEIAKPAKDAVSEVVRSGDLVSYTAEEGVRILGEGKLL
VSDSFPGNERNKYCLSSKVPLGVVLAIPPFNYPVNLAVSKIGPALIAGNSLVLKPPTQGAVAALHMVHCFHLAGFPKGLIS
CVTGKGSEIGDFLTMHPGVNCISFTGGDTGIAISKKAGMVPLQMELGGKDACIVLDDADLDLVAANIVKGGFSYSGQRC
TAVKVVLIMEAVADTVVEKVNAKVAKLKVGPPEDDCDITPVVTESSANFIEGLVMDAKEKGATFCQEYRREGNLIWPLLL
DHVRPDMRIAWEEPFGPVLPVIRINSVEEGIHHCNASNFGLQGCVFTRDINKAIMISDAMESGTVQINSAPARGPDHFPF
QGLKDSGIGSQGITNSINMMTKVKSTVINLPSPSYTMG
>CsGAPDH1
MAGDKKIKIGINGFGRIGRLVARVVLQRDDVELVAVNDPFISTDYMTYMFKYDSVHGQWKHNELKVKDEKTLLFGEKPV
AVFGFRNPEEIPWAKTGAEYVVESTGVFTDKDKAAAHLKGGAKKVVISAPSKDAPMFVVGVNEKEYKPELDIVSNASCTT
NCLAPLAKVIHDKFGIVEGLMTTVHSITATQKTVDGPSMKDWRGGRAASFNIIPSSTGAAKAVGKVLPALNGKLTGMSF
RVPTVDVSVVDLTVRLEKEATYEEIKNAIKEESEGKLKGILGYTEEDVVSTDFVGDSRSSIFDAKAGIALSKNFVKLVSWYDN
EWGYSSRVIDLIVHMAKTQA
>CsGAPDH2
MGKVKIGINGFGRIGRLVARVILQRDDVELVAVNDPFITTDYMTYMFKYDSVHGQWKHHELKVKDDKTLLFGEKPVTVF
GVRNPEEIPWAETGAEYVVESTGVFTDKDKAAAHLKGGAKKVIISAPSKDAPMFVVGVNENEYKPELNIVSNASCTTNCL
APLAKVIHDKFGIVEGLMTTVHSITATQKTVDGPSSKDWRGGRAASFNIIPSSTGAAKAVGKVLPALNGKLTGMAFRVPT
VDVSVVDLTVRLEKDASYDEIKAAIKAESEGKLKGILGYTEDDVVSTDFVGDSRSSIFDAKAGIALSKKFVKLVSWYDNEW
GYSTRVVDLIVHMSKTQ
>CsGAPDH3
MMTRNPEEIPWAETGAELAVESTGVFTDKDKAAAHLKGGTKKVVISAPSKDAPMFVMGVNEKEYKKELDIVSNASCTTT
CLASLAKVIQNKFGIVEGFMTTVHAIAATQKTVDGPSMKDWRRGRAASFNIIPSSDGAAKEYMVDLTVRLEKKATYDDIK
AALKEASQGEMKGIFGYIEDDVVSTDFVGDNRSSIFDAKAGISLSDNLEKLVSWYDTDAAFIFWIGVGILRLRVWSGSGSR
LFT
>CsGAPDH4
MAFSSLLRSTASASLVRADLTSSPSDRVKGSSTAAFSRNLNTSSIFGTSVPSGSSSSSLQTCAAKSIQPIRATATEIPPTIQKS
RSDGNTKVGINGFGRIGRLVLRVAAFRDDVDVVAVNDPFIDAKYMAYMFKYDSTHGVFKGTINVVDDSTLEINGKLIKVF
SKRDPAEIPWGDYGVDYVVESSGVFTTIAKASAHMKGGAKKVVISAPSADAPMFVVGVNEKTYKPNMNIVSNASCTTN
CLAPLAKVVHEEFGILEGLMTTVHATTATQKTVDGPSMKDWRGGRGASQNIIPSSTGAAKAVGKVLPDLNGKLTGMAF

RVPTPNVSVVDLTCRLAKGASYEDVKAAIKYASEGSLKGILGYTDEDVVSNDFVGDSRSSIFDAKAGIGLSASFMKLVSWY
DNEWGYSNRVLDLIEHMALVAAHN
>CsGAPDH5
MASHSALAPSRIPAITRIPSKTTHSFPTQCSTKRLDVAEFAGLRANAGATYATGARDASFFDAVTAQLTPKVAAGSVPVK
KETVAKLKVAINGFGRIGRNFLRCWHGRKDSPLDVVVVNDSGGVKNASHLLKYDSLLGTFKADVKIVDNETISVDGKLIK
VVSNRDPLQLPWAELGIDIVIEGTGVFVDGPGAGKHIQAGAKKVIITAPAKGADIPTYVVGVNEKDYDHEVANIVSNASC
TTNCLAPFVKVMDEELGIVKGAMTTTHSYTGDQRLLDASHRDLRRARAAALNIVPTSTGAAKAVSLVMPQLKGKLNGIA
LRVPTPNVSVVDLVVNVEKKGITAEDVNAAFRKAAEGPLKGILAVCDVPLVSVDFRCSDVSSTIDSSLTMVMGDDMVKV
VAWYDNEWGYSQRVVDLAHLVATKWPGVAAGGSGDPLEDFCQTNPADEECKVYEA
>CsGAPDH6
MASATLSVAKSALQGNGKGFSEFSGLRNSASLPFGRKSSDDFHSVIALQTSALGSSSSGYRKVAAQAKLKVAINGFGRIG
RNFLRCWHGRKDSPLEVVAINDTGGVKQASHLLKYDSTLGIFEADVKPVGTDGISVDGKVIQVVSNRNPVNLPWGDLGI
DLVIEGTGVFVDREGAGKHIQAGAKKVLITAPGKGDIPTYVVGVNADAYKPDEPIISNASCTTNCLAPFVKVLDQKFGIIKG
TMTTTHSYTGDQRLLDASHRDLRRARAAALNIVPTSTGAAKAVALVLPALKGKLNGIALRVPTPNVSVVDLVVQVSKKTF
AEEVNAAFRESADNELKGILSVCDEPLVSVDFRCSDVSSTVDSSLTLVMGDDMVKVIAWYDNEWGYSQRVVDLADIVA
NNWK

GeGAPDH1-DH5A.dna (Linear / 1287 bp)

```
        10        20        30        40        50        60        70
   ATGGCTTCTACCGCTTTCGTAGGATCAGTAACACCCCTTTCCGAAGCTTCCTTCCCTTTTGCGTCAAGCA
1 → ATGGCTTCTACCGCTTTCGTAGGATCAGTAACACCCCTTTCCGAAGCTTCCTTTCCTTTTGCGTCAAGCA
```

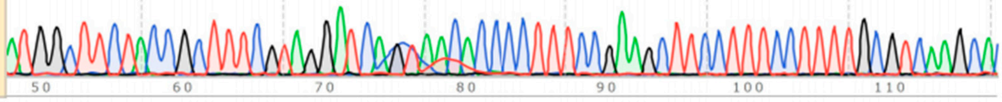

```
        80        90       100       110       120       130       140
   CTCGAGCTCCAGAGCCGTTCAGGGTTTTTTGTTGGCGAAGTAGATCCAGCGAAGGCTCTTCTGTGTTATG
1 → CTCGAGCTCCAGAGCCGTTCAGGGTTTTTTGTTGGCGAAGTAGATCCAGCGAAGGCTCTTCTGTGTTATG
```

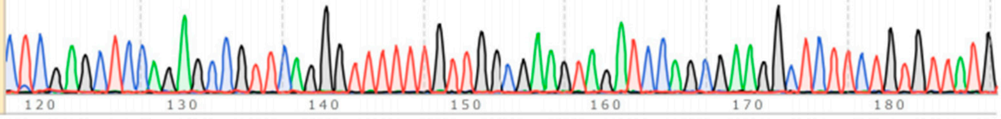

```
       150       160       170       180       190       200       210
   TGCTAGTACTTCATGCTCTAAGTTTCCCTTGGCGGCCATCTCAACACAGAAATCTAATCGTAGAAACTTT
1 → TGCTAGTACTTCATGCTCTAAGTTTCCCTTGGCGGCCATCTCAACACAGAAATCTAATCGTAGAAACTTT
```

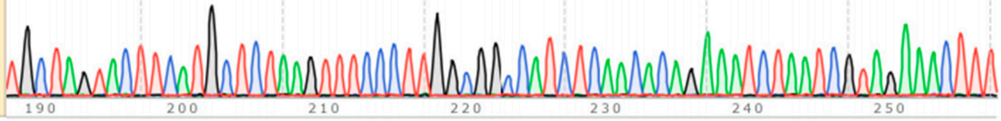

```
       220       230       240       250       260       270       280
   CAGCACACCAAAGCCACAGCAACGGAAACACCTCCTGTTGTTTCTAGATCATCAAGTGATGGGAAGACCA
1 → CAGCACACCAAAGCCACAGCAACGGAAACACCTCCTGTTGTTTCTAGATCATCAAGTGATGGGAAGACCA
```

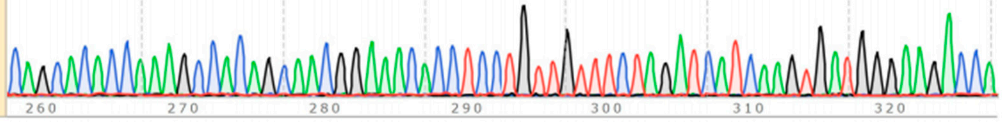

```
       290       300       310       320       330       340       350
   AAGTTGGCATCAATGGTTTTGGTCGTATTGGAAGGTTGGTGTTGCGCATAGCTGCTTCAAGGGATGACAT
1 → AAGTTGGCATCAATGGTTTTGGTCGTATTGGAAGGTTGGTGTTGCGCATAGCTGCTTCAAGGGATGACAT
```

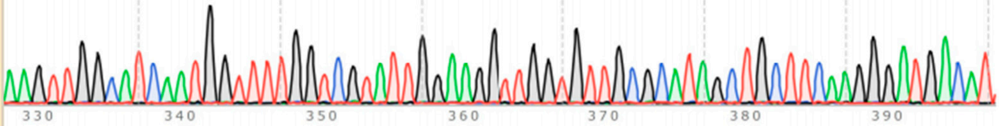

```
2 ←                     GGTCGTATTGGAAGGTTGGTGTTGCGCATAGCTGCTTCAAGGGATGACAT
```

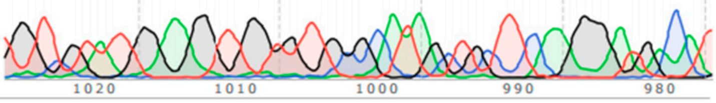

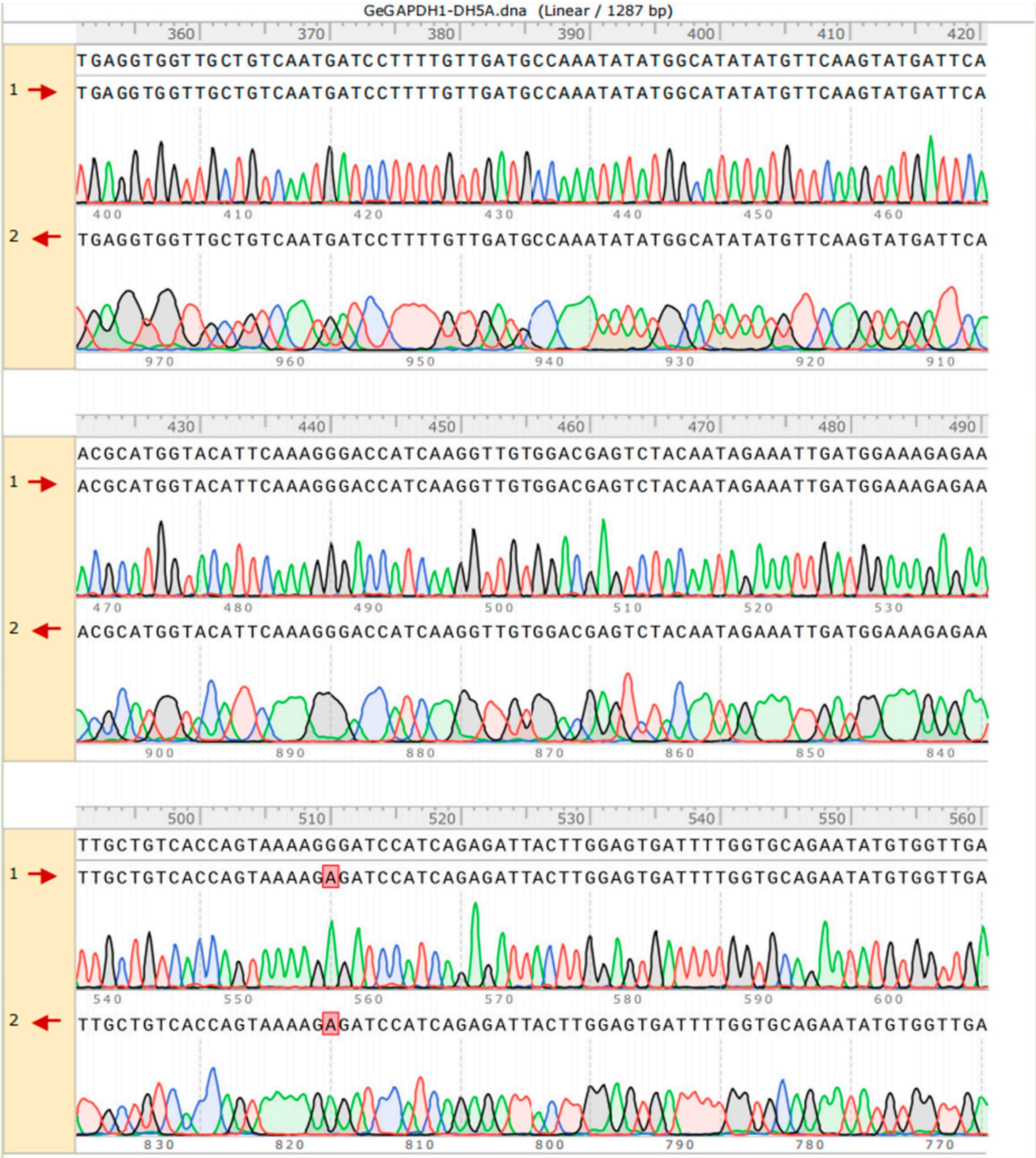
GeGAPDH1-DH5A.dna (Linear / 1287 bp)
TGAGGTGGTTGCTGTCAATGATCCTTTTGTTGATGCCAAATATATGGCATATATGTTCAAGTATGATTCA
1 TGAGGTGGTTGCTGTCAATGATCCTTTTGTTGATGCCAAATATATGGCATATATGTTCAAGTATGATTCA
2 TGAGGTGGTTGCTGTCAATGATCCTTTTGTTGATGCCAAATATATGGCATATATGTTCAAGTATGATTCA
ACGCATGGTACATTCAAAGGGACCATCAAGGTTGTGGACGAGTCTACAATAGAAATTGATGGAAAGAGAA
1 ACGCATGGTACATTCAAAGGGACCATCAAGGTTGTGGACGAGTCTACAATAGAAATTGATGGAAAGAGAA
2 ACGCATGGTACATTCAAAGGGACCATCAAGGTTGTGGACGAGTCTACAATAGAAATTGATGGAAAGAGAA
TTGCTGTCACCAGTAAAAGGGATCCATCAGAGATTACTTGGAGTGATTTTGGTGCAGAATATGTGGTTGA
1 TTGCTGTCACCAGTAAAAGAGATCCATCAGAGATTACTTGGAGTGATTTTGGTGCAGAATATGTGGTTGA
2 TTGCTGTCACCAGTAAAAGAGATCCATCAGAGATTACTTGGAGTGATTTTGGTGCAGAATATGTGGTTGA

GeGAPDH1-DH5A.dna (Linear / 1287 bp)

570 580 590 600 610 620 630

ATCTTCAGGCGTCTTCACAACCATGGACAAAGCATCAGCTCACCTGAAGGGAGGTGCTAAGAAGGTGGTC

1 → ATCTTCAGGCGTCTTCACAACCATGGACAAAGCATCAGCTCACCTGAAGGGAGGTGCTAAGAAGGTGGTC

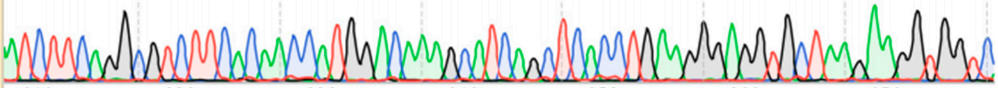

2 ← ATCTTCAGGCGTCTTCACAACCATGGACAAAGCATCAGCTCACCTGAAGGGAGGTGCTAAGAAGGTGGTC

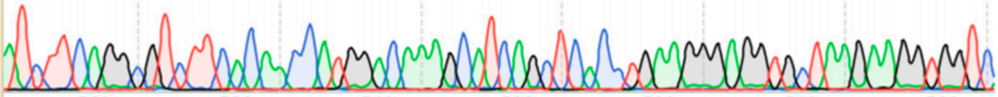

640 650 660 670 680 690 700

ATCTCTGCTCCATCAGCTGACGCCCCTATGTTTGTTGTGGGCGTGAATGAGCAAATGTACCAACCTCACA

1 → ATCTCTGCTCCATCAGCTGACGCCCCTATGTTTGTTGTGGGCGTGAATGAGCAAATGTACCAACCTCACA

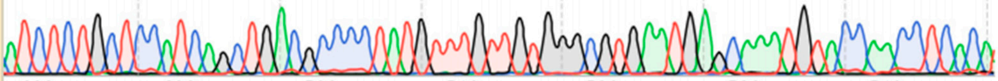

2 ← ATCTCTGCTCCATCAGCTGACGCCCCTATGTTTGTTGTGGGCGTGAATGAGCAAATGTACCAACCTCACA

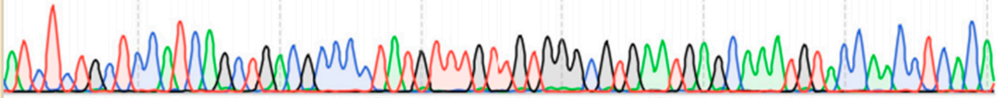

710 720 730 740 750 760 770

TGAACATTGTTTCTAATGCAAGCTGCACCACTAATTGTCTTGCACTATTAGCTAAGGTGGTTCATGAAGA

1 → TGAACATTGTTTCTAATGCAAGCTGCACCACTAATTGTCTTGCACTATTAGCTAAGGTGGTTCATGAAGA

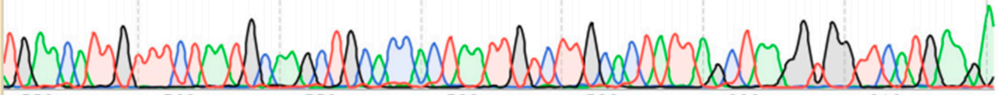

2 ← TGAACATTGTTTCTAATGCAAGCTGCACCACTAATTGTCTTGCACTATTAGCTAAGGTGGTTCATGAAGA

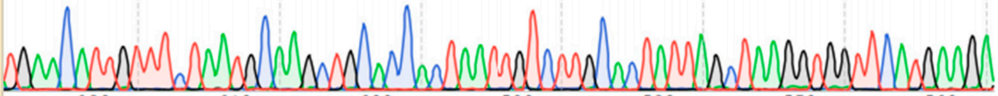

GeGAPDH1-DH5A.dna (Linear / 1287 bp)

780 790 800 810 820 830 840

GTTCGGTATTGTTGAAGGCCTCATGACAACAGTGCATGCAACCACAGCCACACAGAAGACCGTTGATGGC

1 → GTTCGGTATTGTTGAAGGCCTCATGACAACAGTGCATGCAACCACAGCCACACAGAAGACCGTTGATGGC

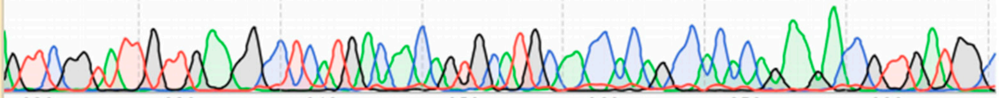

2 ← GTTCGGTATTGTTGAAGGCCTCATGACAACAGTGCATGCAACCACAGCCACACAGAAGACCGTTGATGGC

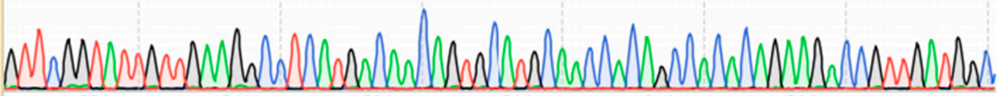

850 860 870 880 890 900 910

CCTTCCATGAAGGACTGGAGAGGGGGCCGCGGAGCCAGCCAGAACATTATCCCTAGCTCAACTGGCGCTG

1 → CCTTCCATGAAGGACTGGAGAGGGGGCCGCGGAGCCAGCCAGAACATTATCCCTAGCTCAACTGGCGCTG

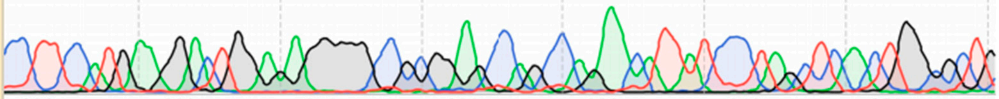

2 ← CCTTCCATGAAGGACTGGAGAGGGGGCCGCGGAGCCAGCCAGAACATTATCCCTAGCTCAACTGGCGCTG

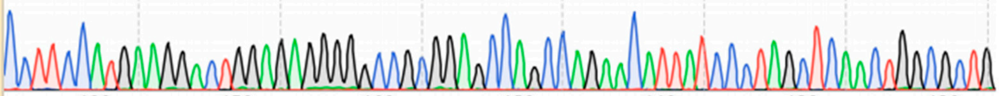

920 930 940 950 960 970 980

CGAAGGCAGTTGGAAAAGTTCTTCCAGAACTCAATGGGAAGCTCACCGGCATGGCTTTTCGGGTCCCAAC

1 → CGAAGGCAGTTGGAAAAGTTCTTCCAGAACTCAATG

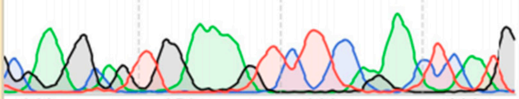

2 ← CGAAGGCAGTTGGAAAAGTTCTTCCAGAACTCAATGGGAAGCTCACCGGCATGGCTTTTCGGGTCCCAAC

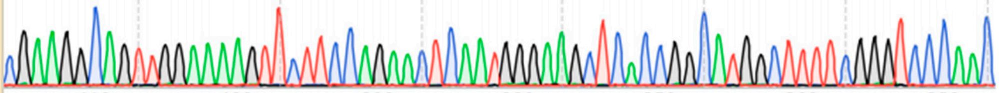

990 1000 1010 1020 1030 1040 1050

AACGAATGTTTCTGTCGTGGACTTGACTTGCCGGCTTGCAAATGATGCGTCCTATGAAGATGTTAAGGCA

2 ← AACGAATGTTTCTGTCGTGGACTTGACTTGCCGGCTTGCAAATGATGCGTCCTATGAAGATGTTAAGGCA

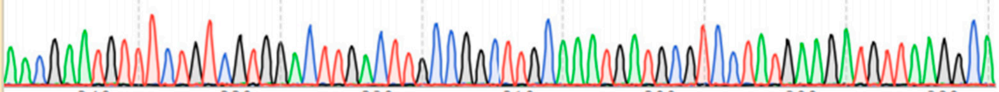

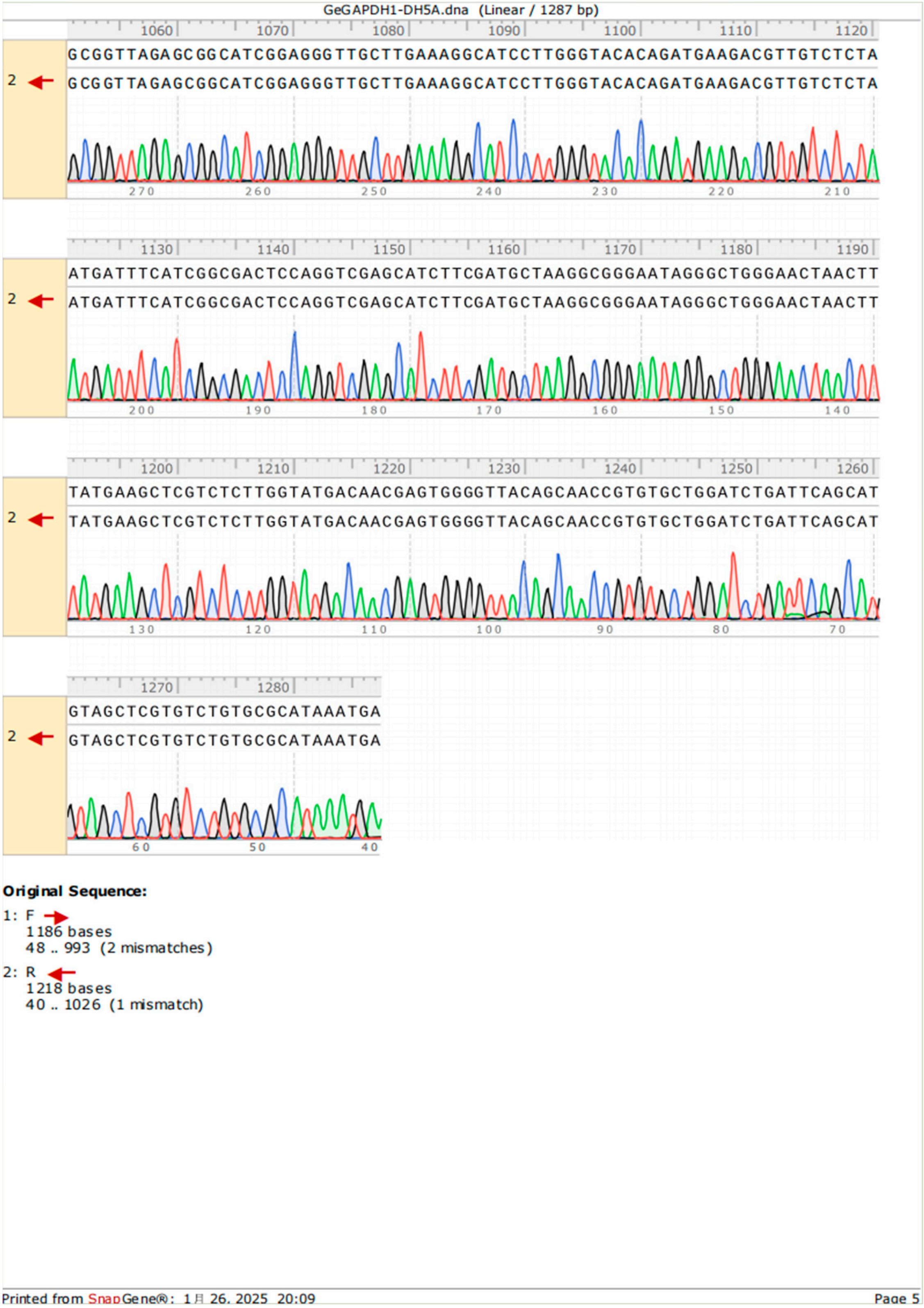

Figure S1. Sequencing results of GeGAPDH1 gene in cloning vector.

The base sequence above represents the sequence from reference genome, and the numbers above it represent the base positions. The base sequence below represents the Sanger sequencing result of gene in cloning vector, and the bottom represents the chromatogram of Sanger sequencing. The results were supported by five single clones.

Sequence: pGEX-4T-1-GeGAPHH1.dna (Linear / 1287 bp)
Enzymes: Unique 6+ Cutters (45 of 653 total) Unique Cutters **Bold**

10 20 30 40 50 60 70 80 90

ATGGCTTCTACCGCTTTCGTAGGATCAGTAACACCCCTTTCCGAAGCTTCCTTCCCTTTTGCGTCAAGCACTCGAGCTCCAGAGCCGTTC

2 → TGGCTTCTACCGCTTTCGTAGGATCAGTAACACCCCTTTCCGAAGCTTCCTTTCCTTTTGCGTCAAGCACTCGAGCTCCAGAGCCGTTC

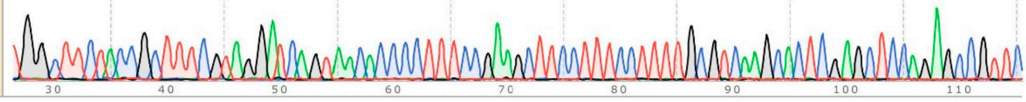

100 110 120 130 140 150 160 170 180

AGGGTTTTTTGTTGGCGAAGTAGATCCAGCGAAGGCTCTTCTGTGTTATGTGCTAGTACTTCATGCTCTAAGTTTCCCTTGGCGGCCATC

2 → AGGGTTTTTTGTTGGCGAAGTAGATCCAGCGAAGGCTCTTCTGTGTTATGTGCTAGTACTTCATGCTCTAAGTTTCCCTTGGCGGCCATC

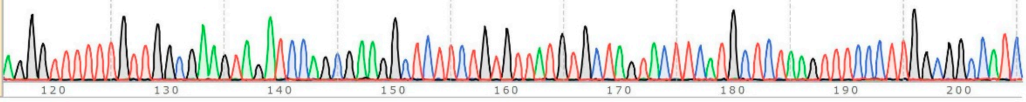

190 200 210 220 230 240 250 260 270

TCAACACAGAAATCTAATCGTAGAAACTTTCAGCACACCAAAGCCACAGCAACGGAAACACCTCCTGTTGTTTCTAGATCATCAAGTGAT

2 → TCAACACAGAAATCTAATCGTAGAAACTTTCAGCACACCAAAGCCACAGCAACGGAAACACCTCCTGTTGTTTCTAGATCATCAAGTGAT

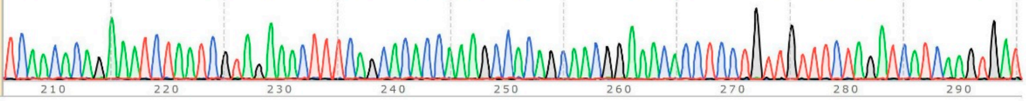

280 290 300 310 320 330 340 350 360

GGGAAGACCAAAGTTGGCATCAATGGTTTTGGTCGTATTGGAAGGTTGGTGTTGCGCATAGCTGCTTCAAGGGATGACATTGAGGTGGTT

1 ← AGCTGCTTCAAGGGATGACATTGAGGTGGTT

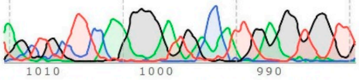

2 → GGGAAGACCAAAGTTGGCATCAATGGTTTTGGTCGTATTGGAAGGTTGGTGTTGCGCATAGCTGCTTCAAGGGATGACATTGAGGTGGTT

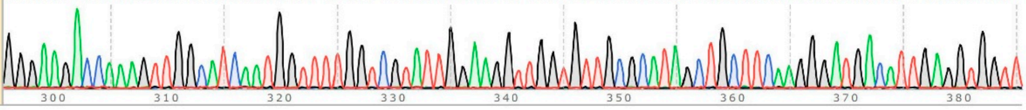

370 380 390 400 410 420 430 440 450

GCTGTCAATGATCCTTTTGTTGATGCCAAATATATGGCATATATGTTCAAGTATGATTCAACGCATGGTACATTCAAAGGGACCATCAAG

1 ← GCTGTCAATGATCCTTTTGTTGATGCCAAATATATGGCATATATGTTCAAGTATGATTCAACGCATGGTACATTCAAAGGGACCATCAAG

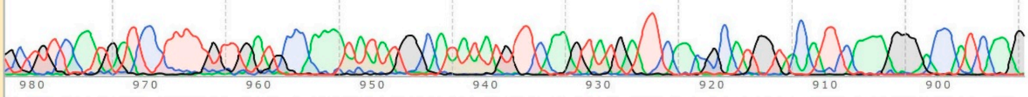

2 → GCTGTCAATGATCCTTTTGTTGATGCCAAATATATGGCATATATGTTCAAGTATGATTCAACGCATGGTACATTCAAAGGGACCATCAAG

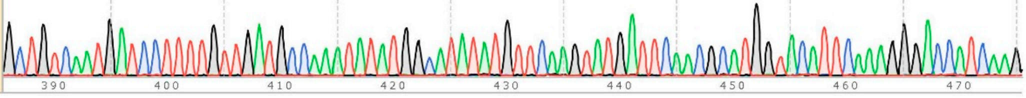

460 470 480 490 500 510 520 530 540

GTTGTGGACGAGTCTACAATAGAAATTGATGGAAAGAGAATTGCTGTCACCAGTAAAAGGGATCCATCAGAGATTACTTGGAGTGATTTT

1 ← GTTGTGGACGAGTCTACAATAGAAATTGATGGAAAGAGAATTGCTGTCACCAGTAAAAGAGATCCATCAGAGATTACTTGGAGTGATTTT

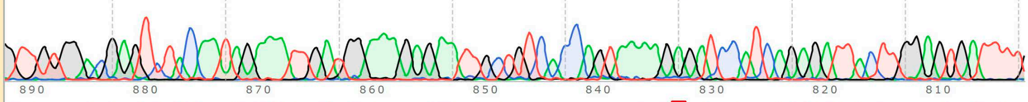

2 → GTTGTGGACGAGTCTACAATAGAAATTGATGGAAAGAGAATTGCTGTCACCAGTAAAAGAGATCCATCAGAGATTACTTGGAGTGATTTT

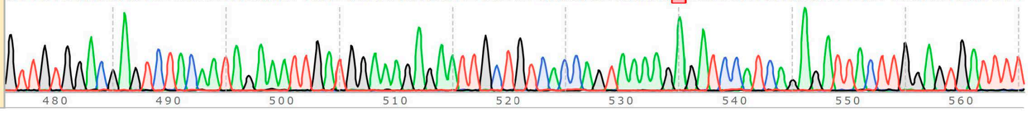

550 560 570 580 590 600 610 620 630

GGTGCAGAATATGTGGTTGAATCTTCAGGCGTCTTCACAACCATGGACAAAGCATCAGCTCACCTGAAGGGAGGTGCTAAGAAGGTGGTC

1 ← GGTGCAGAATATGTGGTTGAATCTTCAGGCGTCTTCACAACCATGGACAAAGCATCAGCTCACCTGAAGGGAGGTGCTAAGAAGGTGGTC

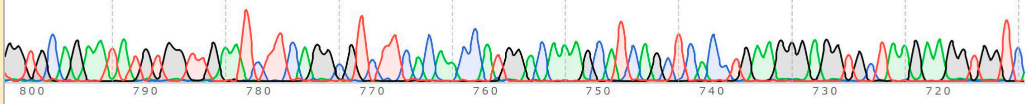

2 → GGTGCAGAATATGTGGTTGAATCTTCAGGCGTCTTCACAACCATGGACAAAGCATCAGCTCACCTGAAGGGAGGTGCTAAGAAGGTGGTC

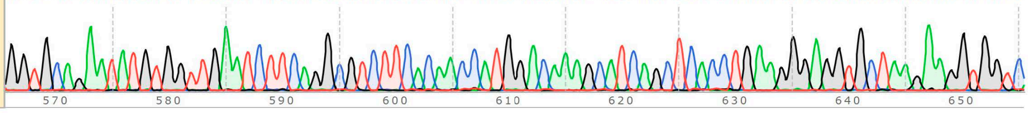

640 650 660 670 680 690 700 710 720

ATCTCTGCTCCATCAGCTGACGCCCCTATGTTTGTTGTGGGCGTGAATGAGCAAATGTACCAACCTCACATGAACATTGTTTCTAATGCA

1 ← ATCTCTGCTCCATCAGCTGACGCCCCTATGTTTGTTGTGGGCGTGAATGAGCAAATGTACCAACCTCACATGAACATTGTTTCTAATGCA

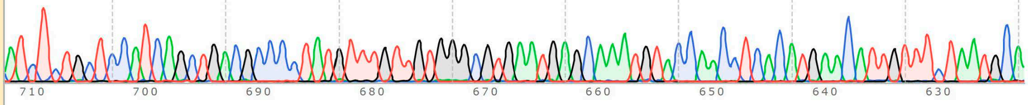

2 → ATCTCTGCTCCATCAGCTGACGCCCCTATGTTTGTTGTGGGCGTGAATGAGCAAATGTACCAACCTCACATGAACATTGTTTCTAATGCA

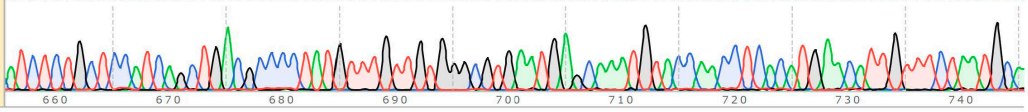

730 740 750 760 770 780 790 800 810

AGCTGCACCACTAATTGTCTTGCACTATTAGCTAAGGTGGTTCATGAAGAGTTCGGTATTGTTGAAGGCCTCATGACAACAGTGCATGCA

1 ← AGCTGCACCACTAATTGTCTTGCACTATTAGCTAAGGTGGTTCATGAAGAGTTCGGTATTGTTGAAGGCCTCATGACAACAGTGCATGCA

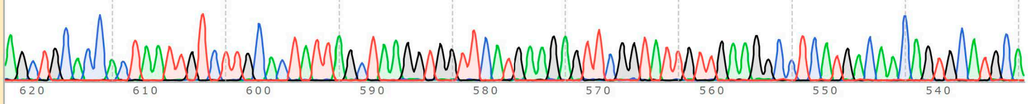

2 → AGCTGCACCACTAATTGTCTTGCACTATTAGCTAAGGTGGTTCATGAAGAGTTCGGTATTGTTGAAGGCCTCATGACAACAGTGCATGCA

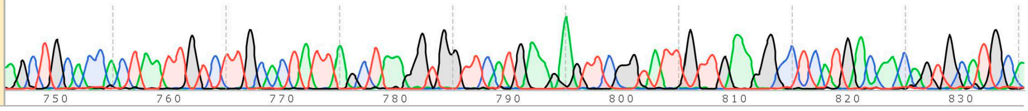

820 830 840 850 860 870 880 890 900

ACCACAGCCACACAGAAGACCGTTGATGGCCCTTCCATGAAGGACTGGAGAGGGGGCCGCGGAGCCAGCCAGAACATTATCCCTAGCTCA

1 ← ACCACAGCCACACAGAAGACCGTTGATGGCCCTTCCATGAAGGACTGGAGAGGGGGCCGCGGAGCCAGCCAGAACATTATCCCTAGCTCA

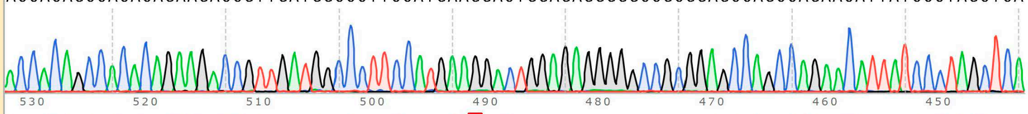

2 → ACCACAGCCACACAGAAGACCGTTGATGGCCCTTCCATGAAAGACTGGAGAGGGGGCCGCGGAGCCAGCCAGAACATTATCCCTAGCTCA

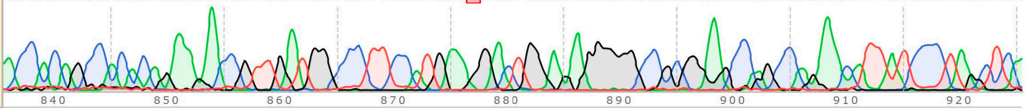

910 920 930 940 950 960 970 980 990

ACTGGCGCTGCGAAGGCAGTTGGAAAAGTTCTTCCAGAACTCAATGGGAAGCTCACCGGCATGGCTTTTCGGGTCCCAACAACGAATGTT

1 ← ACTGGCGCTGCGAAGGCAGTTGGAAAAGTTCTTCCAGAACTCAATGGGAAGCTCACCGGCATGGCTTTTCGGGTCCCAACAACGAATGTT

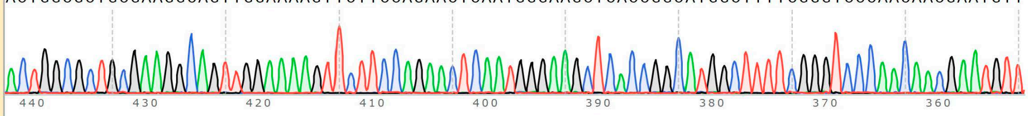

2 → ACTGGCGCTGCGAAGGCAGTTGGAAAAGTTCTTCCAGAACTCAATGGGAAGCTCACCGGCATGGCTTTTCGGGTCCCAACAACGAATGTT

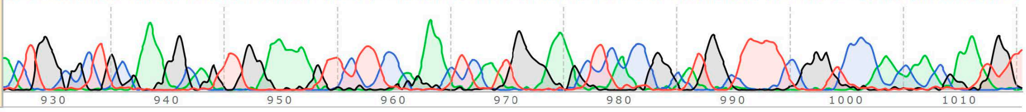

1000 1010 1020 1030 1040 1050 1060 1070 1080

TCTGTCGTGGACTTGACTTGCCGGCTTGCAAATGATGCGTCCTATGAAGATGTTAAGGCAGCGGTTAGAGCGGCATCGGAGGGTTGCTTG

1 ← TCTGTCGTGGACTTGACTTGCCGGCTTGCAAATGATGCGTCCTATGAAGATGTTAAGGCAGCGGTTAGAGCGGCATCGGAGGGTTGCTTG

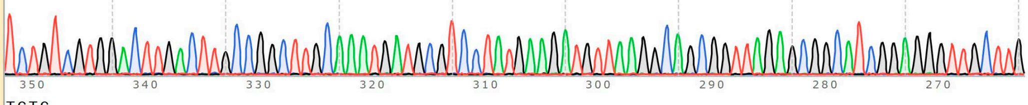

2 → TCTG

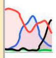

1090 1100 1110 1120 1130 1140 1150 1160 1170

AAAGGCATCCTTGGGTACACAGATGAAGACGTTGTCTCTAATGATTTCATCGGCGACTCCAGGTCGAGCATCTTCGATGCTAAGGCGGGA

1 ← AAAGGCATCCTTGGGTACACAGATGAAGACGTTGTCTCTAATGATTTCATCGGCGACTCCAGGTCGAGCATCTTCGATGCTAAGGCGGGA

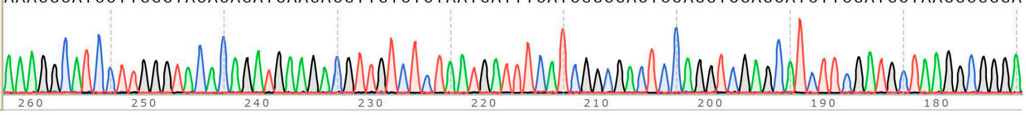

1180 1190 1200 1210 1220 1230 1240 1250 1260

ATAGGGCTGGGAACTAACTTTATGAAGCTCGTCTCTTGGTATGACAACGAGTGGGGTTACAGCAACCGTGTGCTGGATCTGATTCAGCAT

1 ← ATAGGGCTGGGAACTAACTTTATGAAGCTCGTCTCTTGGTATGACAACGAGTGGGGTTACAGCAACCGTGTGCTGGATCTGATTCAGCAT

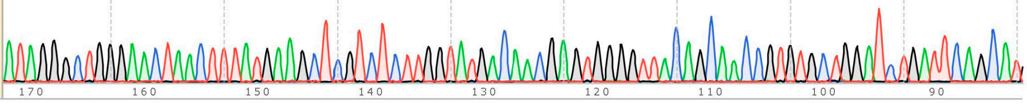

pGEX-4T-1-GeGAPHH1.dna (Linear / 1287 bp)

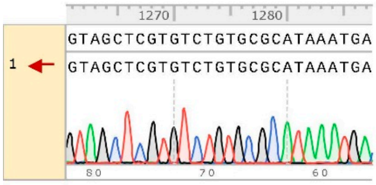

Original Sequence:

1: pGEX3
1013 bases
56 .. 1013 (1 mismatch)

2: pGEX5
1020 bases
27 .. 1019 (3 mismatches)

Printed from SnapGene®: 4月 8, 2025 3:23 Page 4

Figure S2. Sequencing results of GeGAPDH1 gene in expression vector.

The base sequence above represents the sequence from reference genome, and the numbers above it represent the base positions. The base sequence below represents the Sanger sequencing result of gene in expression vector, and the bottom represents the chromatogram of Sanger sequencing. The results were supported by five single clones.

Sequence: pCAMBIA1300-35S-GeGAPDH1-EGFP.dna (Linear / 1287 bp)
Enzymes: Unique 6+ Cutters (45 of 653 total) Unique Cutters **Bold**

10 20 30 40 50 60 70 80 90

ATGGCTTCTACCGCTTTCGTAGGATCAGTAACACCCCTTTCCGAAGCTTCCTTCCCTTTTGCGTCAAGCACTCGAGCTCCAGAGCCGTTC

1 → ATGGCTTCTACCGCTTTCGTAGGATCAGTAACACCCCTTTCCGAAGCTTCCTTCCCTTTTGCGTCAAGCACTCGAGCTCCAGAGCCGTTC

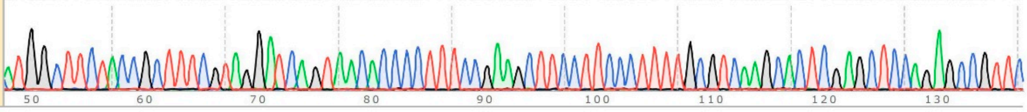

100 110 120 130 140 150 160 170 180

AGGGTTTTTTGTTGGCGAAGTAGATCCAGCGAAGGCTCTTCTGTGTTATGTGCTAGTACTTCATGCTCTAAGTTTCCCTTGGCGGCCATC

1 → AGGGTTTTTTGTTGGCGAAGTAGATCCAGCGAAGGCTCTTCTGTGTTATGTGCTAGTACTTCATGCTCTAAGTTTCCCTTGGCGGCCATC

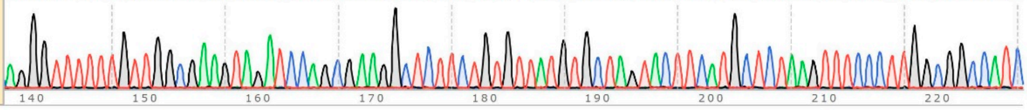

190 200 210 220 230 240 250 260 270

TCAACACAGAAATCTAATCGTAGAAACTTTCAGCACACCAAAGCCACAGCAACGGAAACACCTCCTGTTGTTTCTAGATCATCAAGTGAT

1 → TCAACACAGAAATCTAATCGTAGAAACTTTCAGCACACCAAAGCCACAGCAACGGAAACACCTCCTGTTGTTTCTAGATCATCAAGTGAT

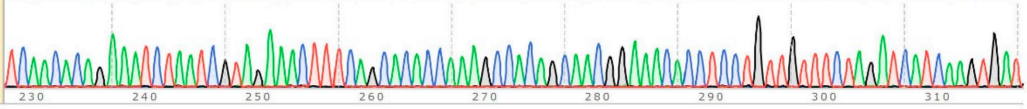

280 290 300 310 320 330 340 350 360

GGGAAGACCAAAGTTGGCATCAATGGTTTTGGTCGTATTGGAAGGTTGGTGTTGCGCATAGCTGCTTCAAGGGATGACATTGAGGTGGTT

1 → GGGAAGACCAAAGTTGGCATCAATGGTTTTGGTCGTATTGGAAGGTTGGTGTTGCGCATAGCTGCTTCAAGGGATGACATTGAGGTGGTT

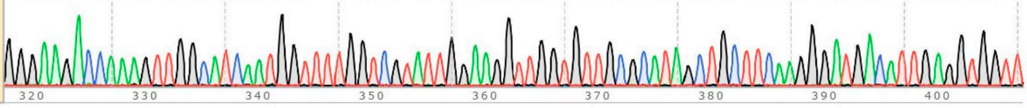

370 380 390 400 410 420 430 440 450

GCTGTCAATGATCCTTTTGTTGATGCCAAATATATGGCATATATGTTCAAGTATGATTCAACGCATGGTACATTCAAAGGGACCATCAAG

1 → GCTGTCAATGATCCTTTTGTTGATGCCAAATATATGGCATATATGTTCAAGTATGATTCAACGCATGGTACATTCAAAGGGACCATCAAG

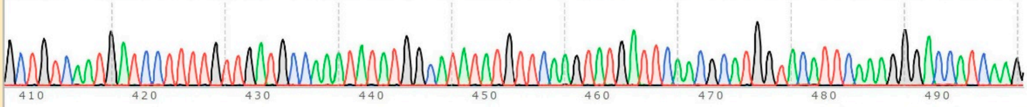

2 ← TTCAAAGGGACCATCAAG

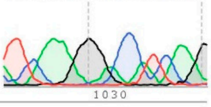

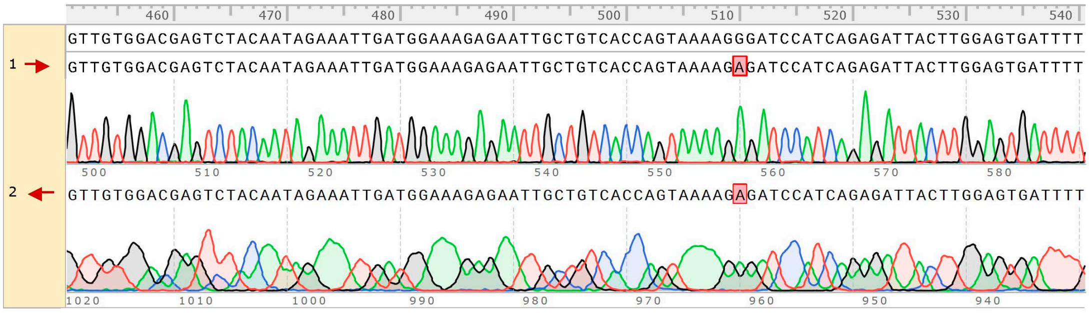
460 470 480 490 500 510 520 530 540
GTTGTGGACGAGTCTACAATAGAAATTGATGGAAAGAGAATTGCTGTCACCAGTAAAAGGGATCCATCAGAGATTACTTGGAGTGATTTT
1
GTTGTGGACGAGTCTACAATAGAAATTGATGGAAAGAGAATTGCTGTCACCAGTAAAAGAGATCCATCAGAGATTACTTGGAGTGATTTT
500 510 520 530 540 550 560 570 580
2
GTTGTGGACGAGTCTACAATAGAAATTGATGGAAAGAGAATTGCTGTCACCAGTAAAAGAGATCCATCAGAGATTACTTGGAGTGATTTT
1020 1010 1000 990 980 970 960 950 940

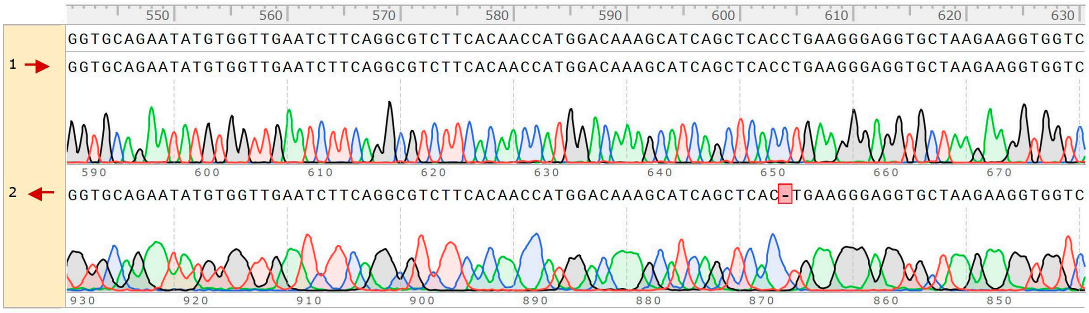
550 560 570 580 590 600 610 620 630
GGTGCAGAATATGTGGTTGAATCTTCAGGCGTCTTCACAACCATGGACAAAGCATCAGCTCACCTGAAGGGAGGTGCTAAGAAGGTGGTC
1
GGTGCAGAATATGTGGTTGAATCTTCAGGCGTCTTCACAACCATGGACAAAGCATCAGCTCACCTGAAGGGAGGTGCTAAGAAGGTGGTC
590 600 610 620 630 640 650 660 670
2
GGTGCAGAATATGTGGTTGAATCTTCAGGCGTCTTCACAACCATGGACAAAGCATCAGCTCAC-TGAAGGGAGGTGCTAAGAAGGTGGTC
930 920 910 900 890 880 870 860 850

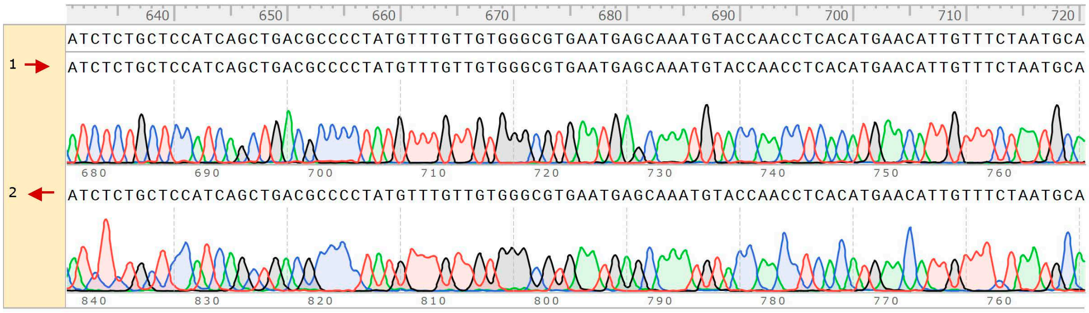
640 650 660 670 680 690 700 710 720
ATCTCTGCTCCATCAGCTGACGCCCCTATGTTTGTTGTGGGCGTGAATGAGCAAATGTACCAACCTCACATGAACATTGTTTCTAATGCA
1
ATCTCTGCTCCATCAGCTGACGCCCCTATGTTTGTTGTGGGCGTGAATGAGCAAATGTACCAACCTCACATGAACATTGTTTCTAATGCA
680 690 700 710 720 730 740 750 760
2
ATCTCTGCTCCATCAGCTGACGCCCCTATGTTTGTTGTGGGCGTGAATGAGCAAATGTACCAACCTCACATGAACATTGTTTCTAATGCA
840 830 820 810 800 790 780 770 760

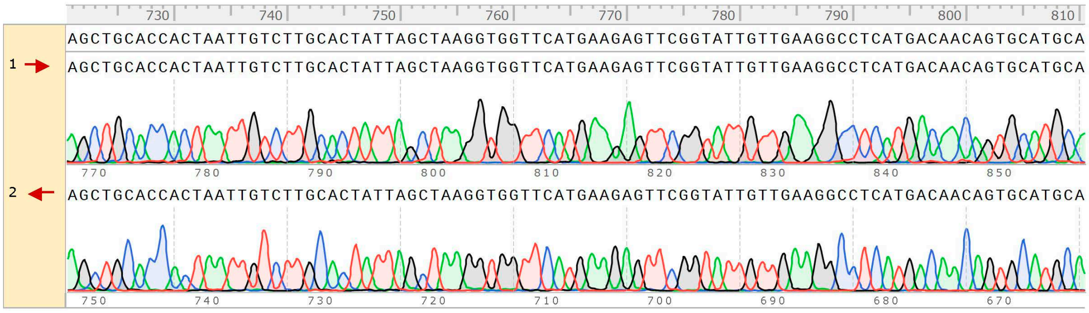
730 740 750 760 770 780 790 800 810
AGCTGCACCACTAATTGTCTTGCACTATTAGCTAAGGTGGTTCATGAAGAGTTCGGTATTGTTGAAGGCCTCATGACAACAGTGCATGCA
1
AGCTGCACCACTAATTGTCTTGCACTATTAGCTAAGGTGGTTCATGAAGAGTTCGGTATTGTTGAAGGCCTCATGACAACAGTGCATGCA
770 780 790 800 810 820 830 840 850
2
AGCTGCACCACTAATTGTCTTGCACTATTAGCTAAGGTGGTTCATGAAGAGTTCGGTATTGTTGAAGGCCTCATGACAACAGTGCATGCA
750 740 730 720 710 700 690 680 670

820 830 840 850 860 870 880 890 900

ACCACAGCCACACAGAAGACCGTTGATGGCCCTTCCATGAAGGACTGGAGAGGGGGCCGCGGAGCCAGCCAGAACATTATCCCTAGCTCA

1 → ACCACAGCCACACAGAAGACCGTTGATGGCCCTTCCATGAAGGACTGGAGAGGGGGCCGCGGAGCCAGCCAGAACATTATCCCTAGCTCA

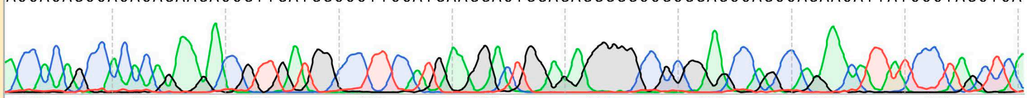

2 ← ACCACAGCCACACAGAAGACCGTTGATGGCCCTTCCATGAAGGACTGGAGAGGGGGCCGCGGAGCCAGCCAGAACATTATCCCTAGCTCA

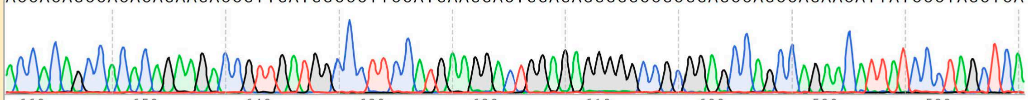

910 920 930 940 950 960 970 980 990

ACTGGCGCTGCGAAGGCAGTTGGAAAAGTTCTTCCAGAACTCAATGGGAAGCTCACCGGCATGGCTTTTCGGGTCCCAACAACGAATGTT

1 → ACTGGCGCTGCGAAGGCAGTTG

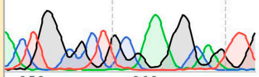

2 ← ACTGGCGCTGCGAAGGCAGTTGGAAAAGTTCTTCCAGAACTCAATGGGAAGCTCACCGGCATGGCTTTTCGGGTCCCAACAACGAATGTT

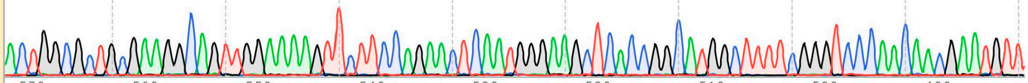

1000 1010 1020 1030 1040 1050 1060 1070 1080

TCTGTCGTGGACTTGACTTGCCGGCTTGCAAATGATGCGTCCTATGAAGATGTTAAGGCAGCGGTTAGAGCGGCATCGGAGGGTTGCTTG

2 ← TCTGTCGTGGACTTGACTTGCCGGCTTGCAAATGATGCGTCCTATGAAGATGTTAAGGCAGCGGTTAGAGCGGCATCGGAGGGTTGCTTG

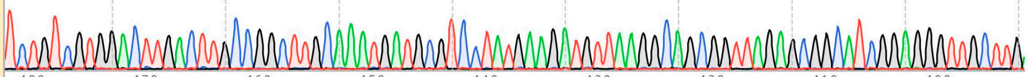

1090 1100 1110 1120 1130 1140 1150 1160 1170

AAAGGCATCCTTGGGTACACAGATGAAGACGTTGTCTCTAATGATTTCATCGGCGACTCCAGGTCGAGCATCTTCGATGCTAAGGCGGGA

2 ← AAAGGCATCCTTGGGTACACAGATGAAGACGTTGTCTCTAATGATTTCATCGGCGACTCCAGGTCGAGCATCTTCGATGCTAAGGCGGGA

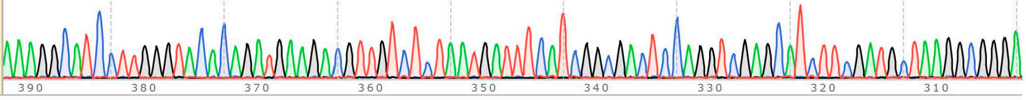

1180 1190 1200 1210 1220 1230 1240 1250 1260

ATAGGGCTGGGAACTAACTTTATGAAGCTCGTCTCTTGGTATGACAACGAGTGGGGTTACAGCAACCGTGTGCTGGATCTGATTCAGCAT

2 ← ATAGGGCTGGGAACTAACTTTATGAAGCTCGTCTCTTGGTATGACAACGAGTGGGGTTACAGCAACCGTGTGCTGGATCTGATTCAGCAT

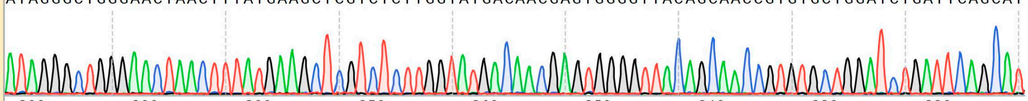

1270 1280

GTAGCTCGTGTCTGTGCGCATAAATGA

2 ← GTAGCTCGTGTCTGTGCGCATAAA

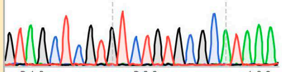

Original Sequence:

1: 1300F →
1138 bases
48 .. 969 (1 mismatch)

2: 1300R ←
1194 bases
189 .. 1039 (1 mismatch, 1 gap)

Figure S3. Sequencing results of GeGAPDH1 gene in subcellular localization vector.

The base sequence above represents the sequence from reference genome, and the numbers above it represent the base positions. The base sequence below represents the Sanger sequencing result of gene in subcellular localization vector, and the bottom represents the chromatogram of Sanger sequencing. The results were supported by five single clones.
